# The gut microbiota reprograms intestinal lipid metabolism through long non-coding RNA *Snhg9*

**DOI:** 10.1126/science.ade0522

**Published:** 2023-08-24

**Authors:** Yuhao Wang, Meng Wang, Jiaxin Chen, Yun Li, Zheng Kuang, Chaitanya Dende, Prithvi Raj, Gabriella Quinn, Zehan Hu, Tarun Srinivasan, Brian Hassell, Kelly Ruhn, Cassie L. Behrendt, Tingbo Liang, Xiaobing Dou, Zhangfa Song, Lora V. Hooper

**Affiliations:** 1 Zhejiang Provincial Key Laboratory of Pancreatic Disease of The First Affiliated Hospital, Institute of Translational Medicine, Zhejiang University School of Medicine, Hangzhou, Zhejiang 310029, China; 2 School of Life Science, Zhejiang Chinese Medical University, Hangzhou, Zhejiang 310053, China; 3 Cancer Center, Zhejiang University, Hangzhou, Zhejiang 310029, China; 4 Department of Immunology, The University of Texas Southwestern Medical Center, Dallas, TX 75390; 5 Department of Breast Surgery and Key Laboratory of Tumor Microenvironment and Immune Therapy of Zhejiang Province, Second Affiliated Hospital, Zhejiang University, Hangzhou, Zhejiang, 310009, China; 6 Department of Colorectal Surgery and Key Laboratory of Biotherapy of Zhejiang Province, Sir Run Shaw Hospital, Zhejiang University, Hangzhou, Zhejiang 310016, China; 7 The Howard Hughes Medical Institute, The University of Texas Southwestern Medical Center, Dallas, TX 75390

## Abstract

The intestinal microbiota promotes lipid absorption and storage by repressing the expression of long non-coding RNA *Snhg9*.

The intestinal microbiota regulates mammalian lipid absorption, metabolism, and storage. Here we report that the microbiota reprograms intestinal lipid metabolism in mice by repressing the expression of long non-coding RNA (lncRNA) *Snhg9* in small intestinal epithelial cells. *Snhg9* suppressed the activity of peroxisome proliferator–activated receptor γ (PPARγ) – a central regulator of lipid metabolism – by dissociating the PPARγ inhibitor Sirtuin 1 from cell cycle and apoptosis protein 2 (CCAR2). Forced expression of *Snhg9* in the intestinal epithelium of conventional mice impaired lipid absorption, reduced body fat, and protected against diet-induced obesity. The microbiota repressed *Snhg9* expression through an immune relay encompassing myeloid cells and group 3 innate lymphoid cells. Our findings thus identify an unanticipated role for a lncRNA in microbial control of host metabolism.

The intestinal microbiota has a significant impact on mammalian metabolism. In mice, resident intestinal bacteria enhance digestion of dietary polysaccharides (1), promote dietary lipid absorption by intestinal epithelial cells (2–5), and alter the function of adipose tissue (6–9). Consistent with the findings in mice, alterations in the composition of the human intestinal microbiota are associated with metabolic disorders including obesity, type 2 diabetes and cardiovascular disease (10–12). Given the rapidly increasing worldwide prevalence of metabolic disease (13), there is a pressing need to understand the mechanisms by which the intestinal microbiota affects host metabolism.

Long non-coding RNAs (lncRNAs) are RNA transcripts that are not translated but are nevertheless biologically functional. LncRNAs regulate biological processes such as cell proliferation, cell death, tumorigenesis, and immunity (14–17), but little is known about their involvement in the regulation of host metabolism by the gut microbiota. We therefore investigated whether the presence of the gut microbiota impacts the transcription of lncRNAs in small intestinal epithelial cells, which are central to microbial modulation of lipid absorption and metabolism (2–5).

## Results

### Expression of lncRNA *Snhg9* is repressed by the microbiota

**Figure 1:**
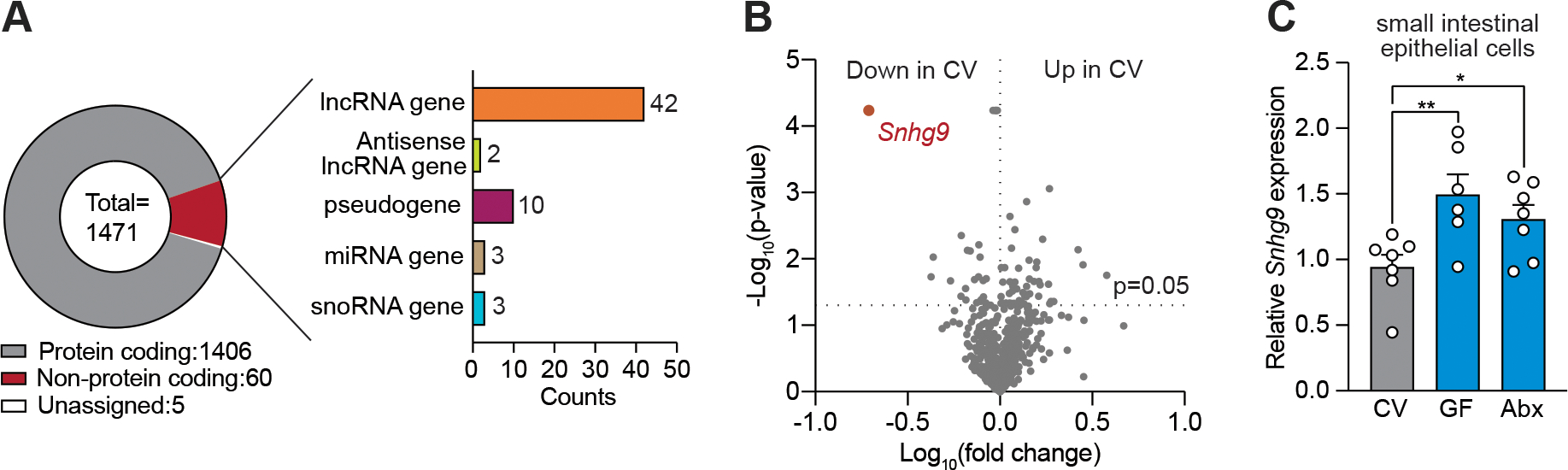
Expression of lncRNA *Snhg9* is repressed by the microbiota. **(A)** Whole transcriptome sequencing of small intestinal epithelial cells recovered by laser capture microdissection from conventional and germ-free mice. Genes differentially expressed between conventional and germ-free mice are summarized and grouped based on transcript type. **(B)** Volcano plot visualizing the changes in lncRNA gene expression between conventional and germ-free mice. *Snhg9* is highlighted in red. **(C)** qPCR analysis of *Snhg9* expression in small intestinal epithelial cells recovered by laser capture microdissection from conventional (CV), germ-free (GF) and antibiotic treated (Abx) mice. Results are representative of at least two independent experiments. Means ± SEM are plotted; each data point represents one mouse. *p<0.05; **p<0.01; two-tailed Student’s *t* test.

Whole transcriptome sequencing (RNA-seq) of small intestinal epithelial cells from conventionally raised (conventional) and germ-free mice identified 60 differentially expressed non-protein coding genes, including 42 genes that encode lncRNAs (Fig. 1A, fig. S1, A and B). In particular, the lncRNA encoded by *Snhg9* (small nucleolar RNA host gene 9) showed reduced abundance in epithelial cells from conventional as compared to germ-free mice (Fig. 1B). Although lncRNAs are generally defined as >200 nucleotides (18) and mouse *Snhg9* RNA is only 183 nucleotides long, human *SNHG9* RNA is 233 nucleotides long and thus both the mouse and human RNAs are designated as lncRNAs (19).

*Snhg9* transcripts were detected in small intestinal epithelial cells of antibiotic-treated mice by in situ hybridization (fig. S2A). Analysis of published single cell RNA sequencing data from mouse intestinal epithelial cells indicated that *Snhg9* transcripts were mostly from stem cells, enterocytes and enteroendocrine cells (a specialized epithelial cell lineage) (20) (fig. S2B). Using quantitative PCR (qPCR), we confirmed that epithelial cells from conventional mice express less *Snhg9* RNA when compared with epithelial cells from both germ-free and antibiotic treated mice (Fig. 1C). Thus, *Snhg9* expression in small intestinal epithelial cells is reduced in the presence of the microbiota.

### LncRNA *Snhg9* binds to CCAR2

**Figure 2:**
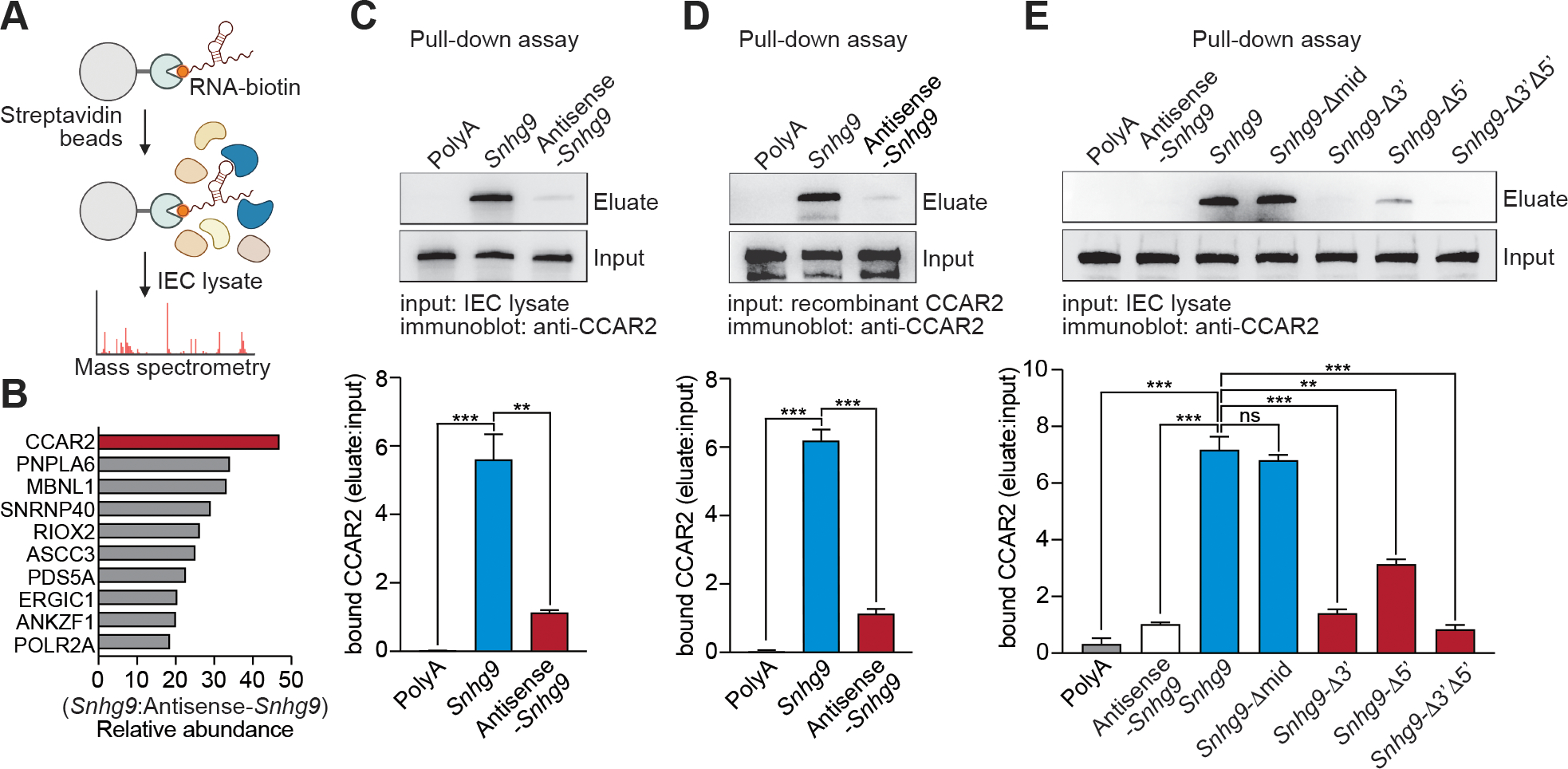
LncRNA *Snhg9* binds to CCAR2. **(A)** Schematic of RNA-protein pull-downs in small intestinal epithelial cell (IEC) lysates in combination with mass spectrometry analysis. Created at BioRender.com. **(B)** Ten most abundant *Snhg9* binding proteins identified by mass spectrometry. CCAR2 is highlighted in red. **(C)** Upper panel: representative immunoblot of CCAR2 in proteins pulled down from small intestinal IEC lysates by polyA RNA (negative control), *Snhg9* or antisense *Snhg9.* Lower panel: Band intensities were quantified by densitometry and normalized to input. N=3 experimental replicates per group. **(D)** Upper panel: representative immunoblot of recombinant CCAR2 pulled down by polyA RNA, *Snhg9* or antisense *Snhg9*. Lower panel: intensities were quantified by densitometry and normalized to input. N=3 experimental replicates per group. **(E)** Upper panel: representative immunoblot of CCAR2 in proteins pulled down from small intestinal IEC lysates by polyA RNA, antisense *Snhg9*, *Snhg9*, *Snhg9* with 28 nucleotides deleted from the middle of the sequence (*Snhg9*-Δmid), *Snhg9* with 3’-deletion of 24 nucleotides (*Snhg9*-Δ3’), *Snhg9* with 5’-deletion of 24 nucleotides (*Snhg9*-Δ5’) or *Snhg9* with both 3’- and 5’-deletion of 24 nucleotides (*Snhg9*-Δ3’Δ5’). Lower panel: band intensities were quantified by densitometry and normalized to input. N=3 experimental replicates per group. All experiments are representative of at least two independent experiments. Means ± SEM are plotted. **p<0.01; ***p<0.001; ns, not significant; two-tailed Student’s *t* test.

We next sought to illuminate the biological function(s) of *Snhg9*. Since other lncRNAs bind to and regulate the activity of proteins (21–23), we screened for *Snhg9*-protein interactions in small intestinal epithelial cells. We performed an RNA-protein pull-down assay in epithelial cell lysates using in vitro transcribed *Snhg9* or antisense *Snhg9*. Mass spectrometry identified the most abundant interacting protein as cell cycle and apoptosis protein 2 (CCAR2; also known as deleted in breast cancer 1, or DBC1) (24) (Fig. 2, A and B). This finding was supported by immunoblot detection of CCAR2 among the proteins precipitated by *Snhg9* (Fig. 2C).

To test whether *Snhg9* binds directly to CCAR2, we performed pull-down assays with in vitro transcribed *Snhg9* and recombinant CCAR2. Consistent with our findings in epithelial cell lysates, recombinant CCAR2 was precipitated by *Snhg9* RNA but not by antisense *Snhg9* RNA or polyA RNA (Fig. 2D), indicating that CCAR2 binds directly to *Snhg9* RNA. In addition, mutations in *Snhg9* impacted binding to CCAR2. Deletion of 28 nucleotides in the middle of the *Snhg9* sequence did not affect *Snhg9* RNA binding to CCAR2. However, binding was reduced by 24-nucleotide deletions in the 3’ and 5’ regions, which target a predicted loop in the *Snhg9* secondary structure (Fig. 2E; fig. S3, A and B). These data support a direct binding interaction and indicate that *Snhg9* is a protein-binding lncRNA that binds directly to CCAR2.

### LncRNA *Snhg9* dissociates CCAR2 from the PPARγ inhibitor SIRT1, repressing PPARγ activity

**Figure 3:**
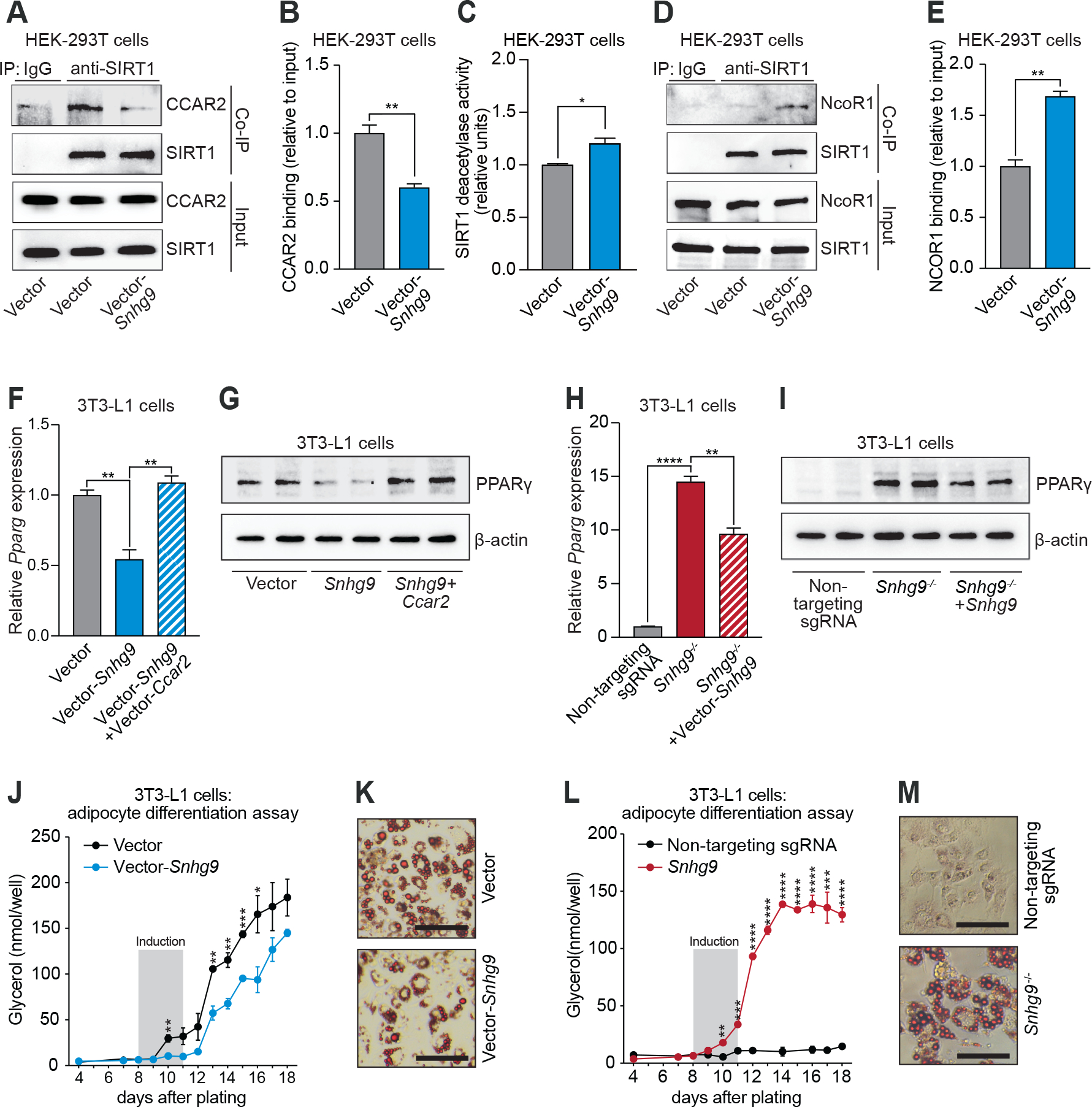
LncRNA *Snhg9* dissociates CCAR2 from the PPARγ inhibitor SIRT1, repressing PPARγ activity. **(A)** Co-immunoprecipitation (co-IP) of CCAR2 and SIRT1 with anti-SIRT1 antibody or IgG isotype control. HEK-293T cells were transfected with empty vector or *Snhg9*-encoding vector. Proteins were detected by immunoblot. **(B)** Band intensities in (A) were quantified by densitometry and normalized to input. N=3 experimental replicates per group. **(C)** Relative SIRT1 deacetylase activity in HEK-293T cells transfected with empty vector or *Snhg9*-encoding vector. N=5 experimental replicates per group. **(D)** Co-immunoprecipitation of NcoR1 and SIRT1 with anti-SIRT1 antibody or IgG isotype control. HEK-293T cells were transfected with empty vector or *Snhg9*-encoding vector. Proteins were detected by immunoblot. **(E)** Band intensities in (D) were quantified by densitometry and normalized to input. N=3 experimental replicates per group. **(F)** qPCR analysis of *Pparg* expression in 3T3-L1 cells with stable expression of *Snhg9* or co-expression of *Snhg9* and *Ccar2*. Cells were transduced with empty vector as a control. N=4 experimental replicates per group. **(G)** Immunoblot detection of PPARγ and β-actin (control) in 3T3-L1 cells from (F). **(H)** qPCR analysis of *Pparg* expression in *Snhg9*^−/−^ 3T3-L1 cells that were untreated or rescued by *Snhg9* expression, and in cells edited with non-targeting sgRNA. N=4 experimental replicates per group. **(I)** Immunoblot detection of PPARγ and β-actin (control) in 3T3-L1 cells from (H). **(J)** *Snhg9* was stably expressed in 3T3-L1 cells and their differentiation to adipocytes was assessed by measuring glycerol as a readout of triglyceride accumulation. Cells were transduced with empty vector as a control. N=5 experimental replicates per group. **(K)** Lipids were detected by Oil Red O staining of differentiated cells from (J). Scale bar=30μm. **(L)** *Snhg9*^−/−^ 3T3-L1 cells and cells edited with non-targeting sgRNA were assessed for differentiation to adipocytes as in (J). N=5 experimental replicates per group. Note that the multiple cell passages required by the CRISPR mutant selection process results in suppression of *Pparg* expression in the cells edited with non-targeting sgRNA (32). **(M)** Lipids were detected by Oil Red O staining of differentiated cells from (L). Scale bar=30 μm. All experiments are representative of at least two independent experiments. Means ± SEM are plotted. *p<0.05; **p<0.01; ***p<0.001; ****p<0.0001; two-tailed Student’s *t* test.

We next investigated the cellular and physiological consequences of lncRNA *Snhg9* binding to CCAR2. CCAR2 is an endogenous inhibitor of the deacetylase Sirtuin 1 (SIRT1) (25, 26). Among its several functions, SIRT1 regulates lipid metabolism by interacting with the transcription factor peroxisome proliferator-activated receptor gamma (PPARγ), which directs transcription of lipid metabolic genes (27–29). SIRT1 represses PPARγ activity through two mechanisms: by deacetylating PPARγ, or by docking with nuclear receptor corepressor 1 (NcoR1), a PPARγ cofactor. Both mechanisms decrease *Pparg* expression and reduce lipid metabolism (30, 31). We reasoned that by binding to CCAR2, *Snhg9* might inhibit its interaction with SIRT1, thereby rescuing SIRT1 deacetylase and NcoR1 binding activities from CCAR2 inhibition. Indeed, overexpression of *Snhg9* in HEK-293T cells largely abolished CCAR2 binding to SIRT1 (Fig. 3, A and B), and increased both SIRT1 deacetylase activity (Fig. 3C) and binding to NcoR1 (Fig. 3, D and E). These data indicate that *Snhg9* promotes SIRT1 activity by sequestering the inhibitory protein CCAR2.

Since *Snhg9* rescued SIRT1 activity from CCAR2 inhibition, we predicted that *Snhg9* would repress PPARγ expression and activity. We therefore evaluated the impact of *Snhg9* on *Pparg* expression and lipid metabolism in cells. PPARγ controls differentiation of the mouse fibroblast cell line 3T3-L1 into adipocytes following chemical induction (30). We generated 3T3-L1 cells that stably express *Snhg9* (fig. S4A) and confirmed binding of *Snhg9* to CCAR2 by RNA immunoprecipitation (RIP) assay (fig. S4B). Stable expression of *Snhg9* inhibited expression of *Pparg* and its protein product PPARγ, whereas co-expression of *Ccar2* rescued expression of both *Pparg* and PPARγ (Fig. 3, F and G). Accordingly, there was reduced transcription of PPARγ-controlled genes, including *Cd36* (encoding a fatty acid transporter), *Fabp4* (encoding a fatty acid binding protein), and *Lpl* (encoding lipoprotein lipase) (fig. S4, C to E). Similarly, stable expression of *Snhg9* in mouse small intestinal organoids reduced expression of *Pparg* and PPARγ-controlled genes (fig. S4, F to J). These effects were not due to expression of the putative *Snhg9* open-reading frame (ORF) (fig. S5A), since expression of the ORF alone failed to reduce expression of *Pparg* and its target genes, and *Snhg9* with mutated start and stop codons retained the ability to reduce *Pparg* expression (fig. S5B). Together, these results indicate that *Snhg9* represses PPARγ expression by binding to CCAR2.

Further supporting this idea, inactivation of *Snhg9* in 3T3-L1 cells (*Snhg9*^*−/−*^ cells) by CRISPR-Cas9 genome editing (fig. S6, A to D) increased expression of *Pparg*, PPARγ, and downstream target genes (Fig. 3, H and I, fig. S6E). This increase occurred despite the high frequency of cell passage during the selection process, which tends to suppress *Pparg* expression. In comparison, cells edited with a non-targeting single guide RNA (sgRNA) showed minimal *Pparg* expression as a result of a high number of cell passages (32) (Fig. 3, H and I), and re-expression of *Snhg9* in *Snhg9*^*−/−*^ 3T3-L1 cells largely reversed *Pparg* expression (Fig. 3, H and I). Of note, overexpression of *Snora78*, which is flanked by the *Snhg9* exons, did not alter *Pparg* expression (fig. S6F), suggesting that *Snora78* deletion did not cause the increased expression of *Pparg* in *Snhg9*^−/−^ cells, and deleting the *Snhg9* locus did not interfere with the expression of a nearby gene, *Rps2* (fig. S6G). These data support the idea that *Snhg9* released SIRT1 from CCAR2 inhibition.

Consistent with the reduced *Pparg* expression, *Snhg9* overexpression restrained the differentiation of 3T3-L1 cells to adipocytes (Fig. 3, J and K). Conversely, *Snhg9*^*−/−*^ 3T3-L1 cells maintained the ability to differentiate and form lipid droplets (Fig. 3, L and M). However, cells edited with non-targeting sgRNA lost the ability to differentiate as a result of insufficient expression of *Pparg* arising from multiple cell passages (32) (Fig. 3, L and M). Taken together, our results reveal that lncRNA *Snhg9* inhibits *Pparg* expression and lipid metabolism by dissociating the CCAR2-SIRT1 complex.

### *Villin-Snhg9* transgenic mice have reduced lipid absorption

The presence of the intestinal microbiota enhances dietary lipid absorption and promotes obesity in mice fed a high fat diet. Germ-free mice, which are microbiologically sterile and thus lack an intestinal microbiota, tend to absorb less lipid than conventional mice and thus are largely protected from high fat diet-induced obesity (2, 3, 7, 8). Since *Snhg9* suppresses lipid metabolism in cells (Fig. 3) and is upregulated in the intestines of germ-free mice (Fig. 1C), we hypothesized that *Snhg9* limits body fat accumulation in mice.

**Figure 4:**
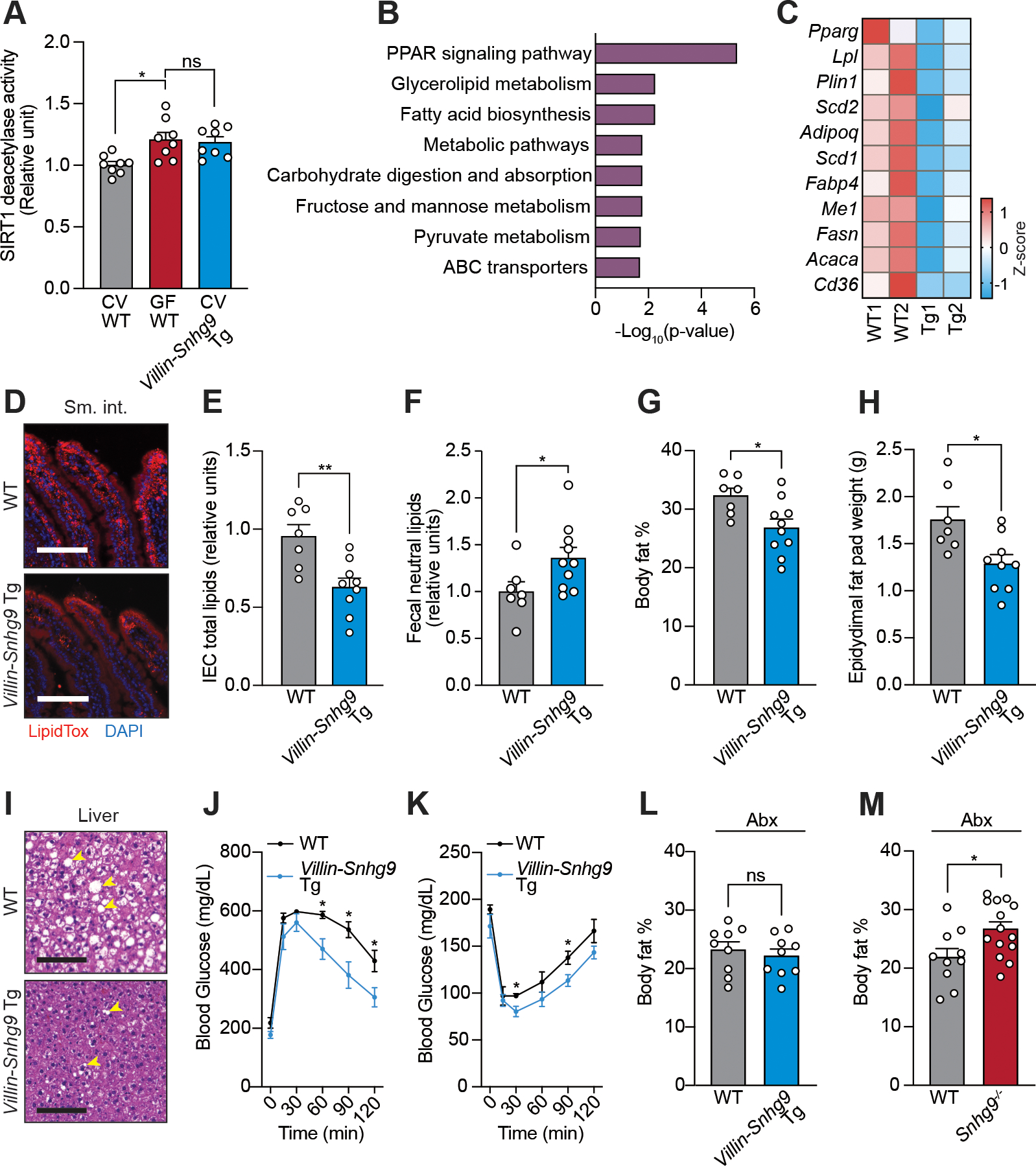
*Villin*-*Snhg9* transgenic mice have reduced lipid absorption and are protected from high fat diet-induced metabolic disorders. **(A)** Relative SIRT1 deacetylation activity in IECs from conventional wild-type, germ-free wild-type and conventional *Villin*-*Snhg9* transgenic (Tg) mice. **(B)** RNA-seq of intestines of wild-type and *Villin*-*Snhg9* Tg littermates. KEGG pathway analysis identifies pathways affected by *Snhg9* overexpression. **(C)** Heatmap visualizing expression levels of selected lipid metabolic genes with altered expression in the small intestines of wild-type (WT) and *Villin*-*Snhg9* Tg littermates. **(D)** LipidTox detection of fatty acids in the small intestines of wild-type and *Villin*-*Snhg9* Tg littermates fed a high fat diet. Scale bar=100 μm. **(E)** Relative total lipid concentrations in isolated IECs from wild-type and *Villin*-*Snhg9* Tg littermates fed a high fat diet. **(F)** Relative total neutral lipid concentrations in the feces of wild-type and *Villin*-*Snhg9* Tg littermates fed a high fat diet. **(G to I)** Wild-type and *Villin*-*Snhg9* Tg littermates were fed a high fat diet for 10 weeks and were assessed for body fat percentage (G), epididymal fat pad weight (H) and liver fat accumulation (examples are indicated with arrowheads) as indicated by hematoxylin and eosin staining (scale bar=100 μm) (I). **(J and K)** Wild-type and *Villin*-*Snhg9* Tg littermates fed a high fat diet were assessed for glucose tolerance (J) and insulin tolerance (K). N=5 mice per group. **(L)** Body fat percentages of wild-type and *Villin*-*Snhg9* Tg littermates that were treated with antibiotics after switching to a high fat diet for 10 weeks. **(M)** Body fat percentages of wild-type and *Snhg9*^*−/−*^ littermates that were treated with antibiotics after switching to a high fat diet for 10 weeks. All experiments are representative of at least two independent experiments. Means ± SEM are plotted; each data point represents one mouse. *p<0.05; **p<0.01; ns, not significant; two-tailed Student’s *t* test.

To test this hypothesis, we used the *Villin* promoter to force expression of *Snhg9* in intestinal epithelial cells of conventional mice (*Villin-Snhg9* transgenic mice; fig. S7, A to D). Consistent with our findings in cultured cells, SIRT1 deacetylase activity was higher in intestinal epithelial cells from conventional *Villin*-*Snhg9* transgenic mice as compared to wild-type littermates, with activity levels comparable to those of wild-type germ-free mice (Fig. 4A).

To investigate whether *Snhg9* overexpression impacts lipid metabolism in vivo, we compared the small intestinal transcriptomes of *Villin*-*Snhg9* transgenic mice and their wild-type littermates by RNA-seq. KEGG pathway analysis confirmed that *Snhg9* overexpression suppressed expression of genes involved in metabolic pathways including the PPAR signaling pathway (Fig. 4B). *Villin*-*Snhg9* transgenic mice showed reduced expression not only of *Pparg* but also genes involved in fatty acid absorption (such as *Cd36*), transport (such as *Fabp4*), and synthesis (such as *Scd1*) (Fig. 4C). Expression of these genes and their protein products was also reduced in germ-free mice in comparison to conventional wild-type mice (fig. S7, E and F). Consequently, *Villin*-*Snhg9* transgenic mice were similar to germ-free mice in that they had less lipid in their intestinal epithelial cells and more in their feces when compared to wild-type littermates (Fig. 4, D to F).

### *Villin-Snhg9* transgenic mice are protected from high fat diet-induced metabolic disorders

When fed a normal chow diet, *Villin*-*Snhg9* transgenic mice had body weights similar to their wild-type littermates (fig. S7G). However, their body fat percentages and epididymal fat pad weights were reduced (fig. S7, H and I) and they were more glucose tolerant (fig. S7J). When switched to a high fat diet for 10 weeks, *Villin*-*Snhg9* transgenic mice exhibited decreased body weights (fig. S7K), lower overall body fat percentages (Fig. 4G), smaller epididymal fat pads (Fig. 4H, fig. S7L) and milder liver steatosis (Fig. 4I) than their wild-type littermates. They also had lower serum triglycerides and free fatty acids (fig. S7, M and N), increased glucose tolerance, and decreased insulin resistance (Fig. 4, J and K). These phenotypes did not result from altered food intake, physical activity, respiratory exchange ratio or microbiota composition (fig. S8, A to C).

Because expression of intestinal *Snhg9* is suppressed by the microbiota (Fig. 1C), we further assessed the requirement for the microbiota in *Snhg9*-regulated lipid metabolism. When we depleted the microbiota by antibiotic treatment, wild-type mice fed a high fat diet had lowered body fat percentages similar to those of *Villin*-*Snhg9* transgenic littermates (Fig. 4L). This is consistent with the increased *Snhg9* expression in antibiotic treated mice (Fig. 1C). For comparison, we generated *Snhg9*^*−/−*^ mice by CRISPR-Cas9-mediated gene targeting (fig. S9, A and B). Although the *Snhg9*^*−/−*^ mice had body weights and body fat percentages similar to wild-type littermates when fed a normal chow diet (fig. S9, C and D), they had increased body fat percentages and weight gain when fed a high fat diet even when their microbiota were depleted with antibiotics (Fig. 4M, fig. S9E). These data support the conclusion that the microbiota promotes body fat accumulation in part by repressing intestinal *Snhg9* expression.

### The microbiota suppresses *Snhg9* expression through a myeloid cell-ILC3 relay

**Figure 5:**
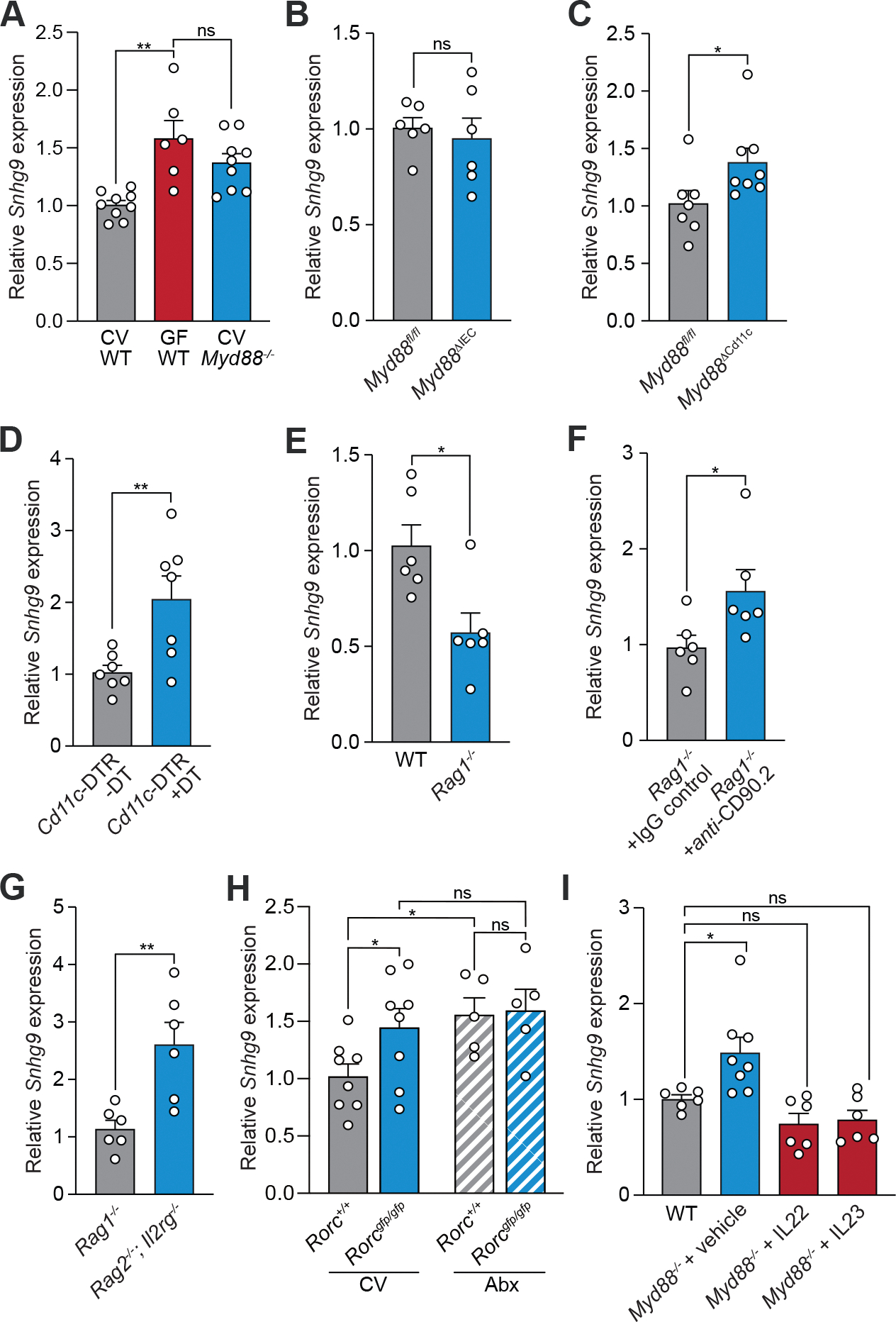
The microbiota suppresses *Snhg9* expression through a myeloid cell-ILC3 relay. *Snhg9* expression was measured by qPCR analysis of the small intestines of **(A)** conventional wild-type, germ-free wild-type and conventional *Myd88*^*−/−*^ mice; **(B)** *Myd88*^*fl/fl*^ and *Myd88*^Δ*IEC*^ (epithelial cell-specific knockout) mice; **(C)** *Myd88*^*fl/fl*^ and *Myd88*^Δ*Cd11c*^ (*Cd11c*^+^ cell-specific knockout) mice; **(D)** *Cd11c*-DTR mice untreated or treated with *Diphtheria* toxin (DT); **(E)** wild-type (WT) and *Rag1*^*−/−*^ mice; **(F)** *Rag1*^*−/−*^ mice injected via the intraperitoneal route with anti-CD90.2 antibody or IgG isotype control; **(G)** *Rag1*^*−/−*^ and *Rag2*^*−/−*^*;Il2rg*^*−/−*^ mice; **(H)** *Rorc*^+/+^ and *Rorc*^*gfp/gfp*^ mice that were untreated (CV) or treated with antibiotics (Abx); **(I)** *Myd88*^*−/−*^ mice treated with recombinant IL-22, IL-23 or vehicle. All experiments are representative of at least two independent experiments. Means ± SEM are plotted; each data point represents one mouse. *p<0.05; **p<0.01; ns, not significant; two-tailed Student’s *t* test.

Bacteria activate intestinal epithelial cell gene expression through Toll-like receptors (TLRs) and their common signaling adaptor MyD88 (33). Expression of intestinal *Snhg9* was increased in *Myd88*^*−/−*^ mice as compared to wild-type controls (Fig. 5A), suggesting that MyD88 is required for microbial repression of intestinal *Snhg9* expression. Although epithelial cell *Myd88* was dispensable for repression of *Snhg9* expression (Fig. 5B), mice with *Myd88* selectively deleted in CD11c^*+*^ cells showed elevated *Snhg9* expression (Fig. 5C), suggesting a role for CD11c^+^ cells in repressing *Snhg9* expression. This idea was supported by studies of a mouse model of CD11c^+^ cell depletion in which *Diphtheria* toxin receptor (DTR) is expressed from the *Cd11c* promoter (34). Selective depletion of CD11c^+^ cells by *Diphtheria* toxin administration increased *Snhg9* expression relative to controls (Fig. 5D). Since CD11c marks myeloid cells, including dendritic cells and macrophages, these results indicate that myeloid cells are required for microbial repression of intestinal *Snhg9* expression.

Bacteria regulate the expression of several key intestinal epithelial cell genes through an immune cell signaling relay involving primarily myeloid cells and group 3 innate lymphoid cells (ILC3) (2, 35–37). In this relay, bacteria activate myeloid cells via TLRs and MyD88, which then signal to ILC3 through the cytokine interleukin 23 (IL-23). Activated ILC3 then signal to intestinal epithelial cells through IL-22 (2, 35–37). Having established a role for myeloid cells in microbial repression of *Snhg9* expression, we next tested for the involvement of ILCs. *Rag1*^*−/−*^ mice, which lack T and B cells, had decreased expression of intestinal *Snhg9* as compared to wild-type mice (Fig. 5E). This indicated that T and B cells are dispensable for the microbial repression of *Snhg9*, while decreased expression of *Snhg9* may be a result of the increased bacterial loads in the intestines of *Rag1*^*−/−*^ mice (36). In contrast, depleting ILCs in *Rag1*^*−/−*^ mice with the CD90.2 antibody (38) elevated *Snhg9* expression (Fig. 5F), indicating a requirement for ILCs in microbial repression of *Snhg9* expression. Similarly, *Rag2*^*−/−*^*;Il2rg*^*−/−*^ mice, which lack immune cells (including ILCs) that dependent on the IL-2 receptor γ chain, showed increased expression of intestinal *Snhg9* as compared to *Rag1*^*−/−*^ mice (Fig. 5G).

To further assess the requirement for ILC3, we analyzed *Snhg9* expression in *Rorc*^*gfp/gfp*^ mice, which lack IL-22 producing cells including ILC3 (38). *Rorc*^*gfp/gfp*^ mice showed increased *Snhg9* expression as compared to wild-type littermates, and antibiotic depletion of gut microbiota abolished this difference (Fig. 5H), supporting the idea that ILC3s relay microbial signals that repress *Snhg9* expression. In addition, supplementing *Myd88*^*−/−*^ mice with either IL-22 or IL-23 repressed *Snhg9* expression to the levels observed in conventional wild-type mice (Fig. 5I), consistent with the known involvement of these cytokines in the myeloid cell-ILC3 signaling circuit (2, 35–37). Further, *Snhg9* expression was repressed by monocolonization of germ-free mice with bacterial species known to activate myeloid cell-ILC3 signaling (2, 35, 37), including *Salmonella enterica* Serovar Typhimurium (*S*. Typhimurium), a Gram-negative intestinal pathogen, and segmented filamentous bacteria (SFB), Gram-positive members of the intestinal microbiota (fig. S10, A and B). Thus, the microbiota suppresses *Snhg9* expression through a myeloid cell-ILC3 signaling relay.

## Discussion and limitations

This study shows that the gut microbiota promotes lipid absorption and metabolism by repressing expression of lncRNA *Snhg9*. This finding raises several additional questions for future study. First, are there specific components of the gut microbiota that promote lipid absorption through *Snhg9* repression? We found that *Snhg9* expression was selectively repressed by bacterial species that activate intestinal myeloid cell-ILC3 signaling, providing a potential clue. However, more studies are needed to determine what bacterial components or characteristics enable activation of myeloid cell-ILC3 signaling, and how microbial community composition impacts *Snhg9* expression and lipid metabolism. Second, what is the evolutionary rationale for microbial regulation of lipid absorption? It is possible that intestinal lipid metabolism is linked to innate immune sensing of microbes in order to cope with an increased energy demand during colonization or infection, to provide lipid substrates or mediators that regulate intestinal immune cell development, or to enhance epithelial barrier function through reactive oxygen species production via lipid oxidation. Third, do these findings provide insight into the regulation of human lipid metabolism? *SNHG9* is conserved and expressed in humans (19), raising the possibility that human *SNHG9* functions in a similar manner.

## Conclusion

In this study, we show that *Snhg9* RNA regulates PPARγ activity by dissociating SIRT1 from CCAR2, providing insight into how a lncRNA regulates intestinal lipid metabolism. These findings advance our understanding of the complex epithelial cell networks that regulate lipid metabolism in response to microbial signals (2, 3)(fig. S11). Ultimately, these results could suggest strategies for treating metabolic disease by targeting *Snhg9* and the microbiota.

## Supplementary Material

Supplementary material

## Data Availability

RNA-seq data and 16*S* rRNA gene sequencing data are available from the Gene Expression Omnibus (GEO) repository under accession number GSE208020. All other data are available in the main text or the supplementary materials.
