# Supplementary material for: The gut microbiota reprograms intestinal lipid metabolism through long non-coding RNA Snhg9
Source: Science. Author manuscript; Available in PMC 2023 Nov 30. (PMC10688608; doi:10.1126/science.ade0522)
Supplement: Supplementary material [file NIHMS1941736-supplement-Supplementary_material.pdf]

Science
AAAS

Supplementary Materials for

The intestinal microbiota reprograms intestinal lipid metabolism through long non-coding RNA *Snhg9*

Yuhao Wang, Meng Wang, Jiaxin Chen, Yun Li, Zheng Kuang, Chaitanya Dende, Prithvi Raj, Gabriella Quinn, Zehan Hu, Tarun Srinivasan, Brian Hassell, Kelly Ruhn, Cassie L. Behrendt, Tingbo Liang, Xiaobing Dou, Zhangfa Song, Lora V. Hooper

Corresponding authors: lora.hooper@utsouthwestern.edu; yuhaowang@zju.edu.cn

This PDF file includes:

Materials and Methods
Figs. S1 to S11
Table S1
References *39-49*

Materials and methods

Mice

C57BL/6 wild-type, *Myd88*$^{-/-}$ (*39*), *Myd88*$^{\Delta IEC}$, *Myd88*$^{\Delta Cd11c}$ (*2*), *Cd11c*-DTR (*33*), *Rag1*$^{-/-}$ (*41*), *Rorc*$^{gfp/gfp}$ (*40*), *Rag2*$^{-/-}$;*Il2rg*$^{-/-}$ (*42, 43*) mice were housed and bred in the SPF barrier at the University of Texas Southwestern Medical Center or at Zhejiang University. *Cd11c*-DTR and *Rorc*$^{gfp/gfp}$ mice were obtained from Jackson Laboratory. *Rag2*$^{-/-}$;*Il2rg*$^{-/-}$ mice were obtained from Taconic Biosciences. The *Villin-Snhg9* transgene was constructed by cloning *Snhg9* cDNA into a p12.4kbVillin-ΔATG plasmid (fig. S7A) (Addgene plasmid #19358; http://n2t.net/addgene:19358; RRID: Addgene_19358). *Villin-Snhg9* Tg mice were generated in the UT Southwestern Transgenic Core by pronuclear injection of the transgene. *Snhg9*$^{-/-}$ mice were generated using CRISPR-Cas9 genome editing with sgRNAs that targeted upstream and downstream of *Snhg9* locus (fig. S6A). sgRNAs were injected into fertilized C57BL/6J embryos along with in vitro transcribed *Cas9* mRNA by the UT Southwestern Transgenic Core. Healthy blastocysts were implanted into pseudo-pregnant mice, and resulting litters were screened by PCR to detect the deletion of the *Snhg9* locus. Germ-free C57BL/6 mice were housed and bred in the gnotobiotic mouse facility at the UT Southwestern Medical Center as described (*44*). 8 to 12 week-old male mice were used for all experiments. All mice were housed under a 12-hour light and 12-hour dark cycle. Mice were fed ad libitum and were euthanized by inhalational anesthetic overdose of isoflurane followed by cervical dislocation. All experiments were performed using protocols approved by the Institutional Animal Care and Use Committees (IACUC) of the UT Southwestern Medical Center and Zhejiang University.

Diet

Mice were fed a normal chow diet (LabDiet 5KA1) containing 22% protein, 16% fat and 62% carbohydrate, or a Western style high-fat diet (Harlan Teklad TD.96132) containing 18.7% protein, 40.6% fat and 40.7% carbohydrate as indicated.

Cell lines

HEK-293T (CRL-11268) and 3T3-L1 (CL-173) cells were obtained from ATCC and were cultured in Dulbecco's Modified Eagle's Medium (DMEM, Thermo Fisher 11965118) supplemented with 10% heat-inactivated fetal bovine serum (FBS) and 10 U/ml Penicillin-Streptomycin (Thermo Fisher 15070063). Cells were cultured in a CO_2 incubator at 37°C.

Antibodies and chemicals

Antibodies were purchased from various vendors: anti-DBC1 antibody (Novus Biologicals NB200-189, Cell Signaling 5857S), anti-SIRT1 antibody (Cell Signaling 8469S, Cell Signaling 9475S, Cell Signaling 3931S), anti-NcoR1 antibody (Cell Signaling 5948S), anti-PPARγ antibody (Cell Signaling 2443S), anti-CD36 antibody (Invitrogen PA5-27236), anti-FABP4 antibody (Cell Signaling S2120S), anti-FASN antibody (Cell Signaling 3180S), anti-β-actin antibody (Cell Signaling 4967S), and anti-CD90.2 antibody (BioLegend 105302). *Diphtheria* toxin (D0564), Flagellin (SRP8029) and lipopolysaccharide (L2012) were purchased from Sigma.

RNA-protein pull-down assay

RNA was transcribed in vitro using a MEGAshortscript T7 transcription Kit (Thermo Fisher AM1354) and purified using a MEGAclear Kit (Thermo Fisher AM1908). 50 pmol purified RNA was denatured at 85ºC for 5 min and then placed on ice immediately. Denatured RNA was labeled with biotinylated cytidine bisphosphate at the 3' end using a Pierce RNA 3' End Desthiobiotinylation Kit (Thermo Fisher 20163) and was purified by phenol:chloroform extraction. 50 µl of streptavidin magnetic beads were washed with 20 mM Tris buffer (pH 7.5) and incubated with 50 pmol biotinylated RNA diluted in RNA capture buffer provided in the Magnetic RNA-protein Pull-down Kit (Thermo Fisher 20164) for 30 min at room

temperature. Beads were then washed with Protein-RNA Binding Buffer (Thermo Fisher 20164) and resuspended in 100 µl RNA-protein binding reaction mixture prepared with 200 µg cell lysate, 50% glycerol and Protein-RNA Binding Buffer. Beads were incubated with cell lysates at 4°C for 1 hour and then washed three times with Wash Buffer (Thermo Fisher 20164). Bound proteins were eluted with 50 µl Elution Buffer (Thermo Fisher 20164) at 37°C for 30 min. Eluted proteins were analyzed by immunoblot or mass spectrometry analysis.

Mass spectrometry

Samples were digested overnight with trypsin following reduction and alkylation with DTT and iodoacetamide. The samples then underwent solid-phase extraction cleanup with an Oasis HLB plate (Waters) and were subsequently dried and reconstituted in 10 ul of 2% acetonitrile, 0.1% trifluoroacetic acid. 5 µl of each sample was injected onto a QExactive HF mass spectrometer coupled to an Ultimate 3000 RSLC-Nano liquid chromatography system. Samples were injected onto a 75 µm inner diameter, 15 cm long EasySpray column (Thermo) and eluted with a gradient from 0-28% buffer B over 90 min with a flow rate of 250 nl/min. Buffer A contained 2% (v/v) acetonitrile and 0.1% formic acid in water, and buffer B contained 80% (v/v) acetonitrile, 10% (v/v) trifluoroethanol, and 0.1% formic acid in water. The mass spectrometer was operated in positive ion mode with a source voltage of 2.4 kV and an ion transfer tube temperature of 275°C. MS scans were acquired at 120,000 resolution in the Orbitrap and up to 20 MS/MS spectra were obtained for each full spectrum acquired using higher-energy collisional dissociation (HCD) for ions with charges 2-8. Dynamic exclusion was set for 20 sec after an ion was selected for fragmentation. Raw data files were analyzed using Proteome Discoverer v2.4 (Thermo), with peptide identification performed using Sequest HT searching against the human protein database from UniProt (downloaded on March 12, 2020). Fragment and precursor tolerances of 10 ppm and 0.02 Da were specified, and three missed cleavages were allowed. Carbamidomethylation of Cys was set as a fixed modification, with oxidation of Met and acetylation of Lys set as a variable modification. The false-discovery rate (FDR) cutoff was 1% for all peptides.

Immunoblot

Cultured cells were washed with PBS and lysed in IP lysis buffer (Thermo Scientific 87788) supplemented with protease inhibitors. Supernatant protein concentrations were measured using the BCA Protein Assay Kit (Thermo Scientific 23225) and normalized across samples. Lysates were separated on 4-20% gradient SDS-PAGE gels and transferred to PVDF membranes. Membranes were blocked with 5% nonfat milk in TBS-T buffer (0.1% Tween-20 in Tris-buffered saline) for 1 hour at room temperature then sequentially incubated with primary antibodies and HRP-conjugated secondary antibodies. Protein bands were visualized using a Bio-Rad ChemiDoc system.

Co-immunoprecipitation

Cultured cells or isolated intestinal epithelial cells were washed with PBS and lysed in IP lysis buffer (Thermo Scientific 87788) supplemented with protease inhibitors. Concentrations of supernatant cell lysates were measured using a BCA Protein Assay Kit (Thermo Fisher 23225). Magnetic protein A/G beads (Thermo Fisher 88803) were washed with IP lysis buffer and incubated with 2 µg of primary antibodies at room temperature for 30 min. Cell lysates with normalized concentrations were added to antibody-bound protein A/G beads and incubated at 4°C for 1 hour with rotation. Beads were then washed with IP lysis buffer four times and boiled in 2X SDS loading buffer at 95°C for 5 min. Eluted proteins were separated on a 4-20% gradient SDS-PAGE gel and transferred to a PVDF membrane. The membrane was sequentially incubated with primary antibodies and appropriate HRP-conjugated secondary antibodies. Protein bands were visualized using a Bio-Rad ChemiDoc system.

RNA immunoprecipitation (RIP)

RNA immunoprecipitation assays were performed using a Magna RIP RNA-binding Protein Immunoprecipitation kit (Millipore 17-700). In brief, 3T3-L1 cells which stably express *Snhg9* were washed with cold PBS and lysed in RIP Lysis Buffer supplemented with protease inhibitor and Rnase inhibitor. Cell lysate supernatants were added to magnetic protein A/G beads bound with anti-CCAR2 antibody or IgG isotype control antibody and incubated overnight at 4°C with rotation. Beads were thoroughly washed with cold RIP Wash Buffer, then incubated in RIP Elution Buffer supplemented with 1% SDS and 100 µg/ml proteinase K at 55°C for 30 min with shaking to elute RNA. Eluted RNA was purified using phenol:chloroform:isoamyl alcohol and then precipitated by ethanol. RNA pellets were air-dried and resuspended in RNase-free water.

In situ hybridization

Digoxigenin (DIG)-labeled antisense or sense probes were synthesized by the HiScribe T7 Quick High Yield RNA Synthesis Kit (NEB E2050S). Sections of paraffin-embedded ileum tissue were washed twice with xylene followed by rehydration in a series of ethanol solutions. Ileum sections were permeabilized with 20 µg/ml of proteinase K for 10 min then treated with 20% ice-cold acetic acid for 20 seconds. The sections were prehybridized with 50% formamide, 5X saline sodium citrate, 1 mg/ml of yeast tRNA (Roche 10109517001), 0.1 mg/ml of heparin (Sigma H3149), 1X Denhardt's solution (Sigma D2532), and 0.1% Tween-20 at 60°C for two hours and then hybridized with synthesized probe (0.5 mg/ml) at 60°C for 16 hours. After washing, sections were incubated with peroxidase-conjugated anti-DIG antibody (Roche 11207733910), followed by biotin-labeled tyramide (ThermoFisher B40951) for signal amplification. Hybridized probes were detected by ABC-Alkaline phosphatase (Vector Laboratories AK-5000) and NBT/BCIP (Roche 11681451001). Images were captured using a Leica DM4000 microscope.

Generation of *Snhg9*$^{-/-}$ 3T3-L1 cells

sgRNAs targeting regions upstream and downstream of *Snhg9* locus (fig. S6A) were cloned into LentiCRISPR v2 plasmid. Mammalian lentiviral particles harboring sgRNA-encoding plasmids were generated in HEK293T cells transfected with LentiCRISPR v2. After transduction and puromycin selection, single 3T3-L1 cells were seeded into each well of a 96-well plate by serial dilution. Positive clones were screened by PCR and verified by Sanger sequencing. Control 3T3-L1 cells were generated through the same procedure except using a non-targeting sgRNA (*45*). LentiCRISPR v2 plasmid was from Dr. Feng Zhang (Addgene plasmid #52961; http://n2t.net/addgene:52961; RRID: Addgene_52961). sgRNA oligo sequences are given in Table S1.

SIRT1 activity assay

Cultured cells or isolated intestinal epithelial cells were washed with PBS and lysed in IP lysis buffer (Thermo Scientific 87788) supplemented with protease inhibitors. Cell lysates with normalized protein concentrations were added to Protein G-agarose beads bound with SIRT1 antibody. Beads were incubated for one hour at 4°C with rotation then sequentially washed with IP lysis buffer and SIRT1 assay buffer (50 mM Tris pH 8.8, 0.5 mM DTT). Beads were resuspended in SIRT1 assay buffer and SIRT1 activity measurements were conducted using a SIRT1 Activity Assay Kit (Abcam ab156065).

Intestinal organoid culture

Intestinal crypts were isolated from mouse ileum using Gentle Cell Dissociation Reagent (StemCell 07174) and filtered through a 70 µm cell strainer. Isolated crypts were resuspended in complete IntestiCult Organoid Growth Medium (StemCell 06005) together with an equal volume of Matrigel Matrix (Corning 354277). 50 µl of crypt suspension was seeded onto the center of each well of a 24-well plate. The plate was incubated at 37°C for 10 min to set the Matrigel and then 600 µl of complete IntestiCult Organoid

Growth Medium was added to each well. Crypts were grown at 37°C for organoid growth and organoid cultures were passaged every 7-10 days.

Laser capture microdissection and RNA purification

Laser capture microdissection was performed as described (*46*). In brief, ~5 cm of distal mouse ileum was washed and snap-frozen in optimum cutting temperature (OCT) compound (Sakura 4583). A 7 µm frozen section was cut, mounted on a glass slide, fixed in 70% ethanol, and then sequentially stained with methyl green and eosin. Freshly stained sections were immediately used for laser capture microdissection of intestinal epithelial cells using an Arcturus PixCell II system, with 5,000-10,000 pulses obtained from each section. Total RNA was extracted and purified using PicoPure RNA Isolation Kit (Qiagen) following the manufacturer's protocol. RNA quality and concentration were determined on an Agilent 2100 Bioanalyzer or RiboGreen S2 RNA Assay Kit (Thermo Fisher R11490). Total RNA from whole tissue was isolated and purified using a Rneasy Midi Kit (Qiagen 75144).

Quantitative real-time PCR (qPCR)

cDNA was synthesized from purified RNA using M-MLV Reverse Transcriptase (ThermoFisher 28025021). qPCR was performed using the Platinum SYBR Green qPCR SuperMix-UDG (Thermo Fisher 11733046) on a QuantStudio 7 Flex Real-Time PCR System (Applied Biosystems). Relative expression values were determined using the comparative Ct (ΔΔCt) method (*47*), and transcript abundances were normalized to *Gapdh* transcript abundance. Primer sequences are given in Table S1.

Differentiation of 3T3-L1 cells and adipogenesis assay

2×10^4 3T3-L1 cells were seeded into each well of a 96-well plate. Cells were cultured in complete DMEM medium until confluent. Differentiation was initiated by switching culture medium to complete DMEM/F-12 medium containing 1.5 µg/ml insulin, 1 µM dexamethasone, 500 µM IBMX and 1 µM rosiglitazone (BioVision K579). Cells were cultured in differentiation medium for three days then switched to complete DMEM/F-12 medium containing 1.5 µg/ ml insulin. Cells were maintained in maintenance medium until fully differentiated. For the adipogenesis assay, 3T3-L1 cells were collected at different time points during the differentiation process. Adipogenesis assays were performed using an Adipogenesis Colorimetric/ Fluorometric Assay Kit (BioVision K610).

Oil Red O staining

Cultured and differentiated 3T3-L1 cells were washed with phosphate buffered saline (PBS) and fixed in 10% buffered formalin at room temperature. Fixed cells were washed with water and rinsed with 60% isopropanol, then stained with freshly prepared Oil Red O (Sigma O0625) working solution (60% of 0.5 g/100 ml Oil Red O stock solution dissolved in isopropanol, 40% distilled water) for 10 min at room temperature. Stained cells were washed in 60% isopropanol followed by three changes of water. Images were captured using a Keyence BZ-X microscope.

Metabolic studies

Mouse body fat percentage was measured with an EchoMRI-100H analyzer. Blood glucose was measured using a One Touch Ultra2 glucose meter. Serum triglycerides were quantified using Infinity Triglycerides Liquid Stable Reagent (Thermo Scientific TR22421). Free fatty acids were measured using the Wako NEFA-HR (2) reagent (Wako 434-91795, 436-91995, 270-77000). Serum triglycerides, free fatty acids, and glucose tolerance were measured after an overnight fast. The insulin tolerance test was performed after a 4-hour fast. Mice were injected intraperitoneally with 2 mg/g body weight of D-(+)-glucose (Sigma G8769), or 0.5 U/kg body weight insulin (Eli Lilly, Humulin R) and blood glucose was measured at 0, 15, 30, 60 and 100 min after injection. Real-time metabolic cage analysis was performed with the TSE Labmaster System.

LipidTox staining

Mouse intestines were cut and prefixed in fresh 4% paraformaldehyde (PFA) for at least two hours at room temperature, then soaked and washed three times in 2% sucrose-PBS solution. Prefixed tissue was snap frozen in OCT compound (Sakura) and 7 μm sections were cut and mounted on glass slides. Slides were fixed in 4% fresh PFA for 20 min and washed twice with PBS. Fixed slides were stained for 30 min in the dark with LipidTox (Thermo Fisher H34476) working solution (1:200 diluted in PBS), followed by staining for 10 min with 4′,6-diamidino-2-phenylindole (DAPI; 1 μg/ml). Images were captured using a Keyence BZ-X microscope.

Hematoxylin & Eosin (H&E) staining

Mouse liver was cut and fixed in 10% buffered formalin overnight at 4ºC, then thoroughly washed with PBS. Fixed liver was paraffin embedded and then 7 μm sections were cut and mounted on glass slides. Slides were sequentially stained with hematoxylin and eosin. Images were captured using a Keyence BZ-X microscope.

Lipid quantification in intestinal epithelial cells

Intestinal epithelial cells were isolated from mouse ileum using 10 mM EDTA (*2*). 10 mg (wet weight) epithelial cells were used for lipid extraction using a Lipid Extraction Kit (Cell Biolabs STA-162). Extracted lipids were air dried, resuspended in 50 μl cyclohexane, and quantified using a Lipid Quantification Kit (Cell Biolabs STA-613).

Neutral lipid quantification in fecal pellets

Fresh fecal pellets were collected and weighed. Fecal lipids were extracted using a Lipid Extraction Kit (Cell Biolabs STA-162). Extracted lipids were air dried, resuspended in 100 μl isopropanol, and quantified using a Lipid Quantification Kit for neutral lipids (Cell Biolabs STA-617).

Antibiotic treatment of mice

Conventionally raised C57BL/6 mice were first gavaged with 200 μl of antibiotic cocktail water containing 2 mg/ml neomycin, 2 mg/ml gentamycin, 2 mg/ml metronidazole, 2 mg/ml streptomycin and 1 mg/ml vancomycin. Mice were maintained on drinking water containing 1 mg/ml neomycin, 1 mg/ml gentamycin, 1 mg/ml metronidazole, 1 mg/ml streptomycin and 0.5 mg/ml vancomycin for at least seven days or throughout the experiment. Microbiota depletion was verified by aerobic and anaerobic culture of fecal pellets.

Recombinant IL-23 and IL-22 treatment

Myd88$^{-/-}$ mice were injected intraperitoneally with 1.5 μg of carrier-free recombinant mouse IL-23 (BioLegend 589006) or IL-22 (BioLegend 576206) every other day for a total of four treatments. Mice were sacrificed on the day after the last injection.

Monocolonization of germ-free mice and flagellin/LPS challenge

Germ-free mice were colonized with 2×10^9 CFU of log phage *Bacteroides thetaiotaomicron* (VPI-5482) or *Enterococcus faecalis* (ATCC29212), or with 1×10^9 CFU log phase *Salmonella enterica* Serovar Typhimurium (strain 1433) through oral gavage. Mice were sacrificed three days after colonization. For SFB monocolonization, fecal material from SFB-monocolonized mice (obtained from Andrew Gewirtz's lab at the Georgia State University) was resuspended in 1 ml PBS. The suspension was centrifuged at 1000*g* and germ-free mice were gavaged with 100 μl of the supernatant. Mice were sacrificed four weeks after colonization. For flagellin challenge, germ-free mice were given 2 μg flagellin every day for three days delivered through the retro-orbital vein and were sacrificed the following day after the last injection. For

lipopolysaccharide (LPS) challenge, germ-free mice were given 500 µg LPS every 12 hours for three days through oral gavage and were sacrificed 12 hours after the last treatment.

Transgenic mouse whole genome sequencing and data analysis

High molecular weight genomic DNA was extracted from mouse tail tissue using the Qiagen DNA extraction kit (Qiagen 6060). A high complexity sequencing library was constructed using Illumina DNA prep M Tagmentation (Illumina 20018705). The quality and quantity of the final library was assessed using Bioanalyzer 2100 and qPCR, respectively. The quality pass library was sequenced on NovaSeq6K S4 lane using a paired-end 150 bp sequencing kit. To detect reads originating from the transgene, the full sequence of linearized p12.4kb-*Villin* vector with *Snhg9* cloned in was used to create a custom mouse genome reference (mm10 plus transgene, https://www.addgene.org/19358/sequences/). Reads generated on a NovaSeq 6000 (SP4 flow cell, PE150 run format) were mapped to the custom reference using Map Reads to Reference tool (CLC Genomics Workbench, version 21.0.4), followed by calling structural variants (i.e. deletions and insertions). Full length paired reads that mapped entirely to the host or entirely to the transgene were filtered out. In addition, we created a custom BLAST database that included mm10 plus the transgene sequences and used the reads as queries (BLAST and Create BLAST Database tools, CLC Genomics Workbench, version 21.0.4). Sequences that had strong hits to the transgene were identified by their unique read IDs and searched in the mapped reads, and accuracy of their genomic positions were further ranked by combining both the mapping quality and BLAST scores.

Single-cell RNA sequencing data analysis

Gene count matrices from GSE195742 were filtered for cells that had greater than 600 unique features, greater than 1000 read counts, and less than half of all reads belonging to mitochondrial genes. Reads from filtered cells were log normalized, associations with the percentage of mitochondrial genes were regressed out, and dimension reduction was performed. Cells were annotated by marker genes highlighted in the original publication (*20*). All analyses were performed using Seurat R Package on the UTSouthwestern BioHPC platform.

RNA sequencing and data analysis

RNA was extracted and purified from isolated mouse intestinal epithelial cells or whole ileum. RNA quality was assessed on an Agilent 2100 Bioanalyzer. Sequencing libraries were prepared using the TruSeq RNA sample preparation kit v2 (Illumina). Sequencing was performed on an Illumina HiSeq 2500 for single end 50 bp length reads. Sequence data were mapped against the mm10 genome using TopHat and FPKMs were generated using Cuffdiff (*48*) with default parameters. Altered expression was defined as a >2-fold increase or decrease in average FPKM reads compared between the two groups. Gene feature types were annotated according to the Mouse Genome Informatics (MGI) database developed by the Jackson Laboratory. KEGG pathway analysis was performed using WEB-based Gene Set Analysis Toolkit (WebGestalt).

16*S* rRNA gene sequencing and data analysis

Fecal DNA was extracted and purified from freshly collected feces using the FastDNA Spin Kit (MP Biomedicals 116560-200) and a FastPrep-24 5G Homogenizer. Sequencing libraries were prepared using the HotStarTaq Plus Master Mix Kit (Qiagen) with primers flanking variable regions V3-V4. Sequencing was performed on a MiSeq following the manufacturer's guidelines. Operational taxonomic units (OTUs) were defined by clustering at 3% divergence (97% similarity). Final OTUs were taxonomically classified using BLASTn against a curated database derived from RDPII and NCBI.

Statistics

All data are shown as means ± standard error of the mean (SEM). Statistical analysis was performed using a two-tailed Student's *t*-test. For all tests, p values lower than 0.05 were considered statistically significant. Details of statistical analysis for each experiment are described in the figure legends.

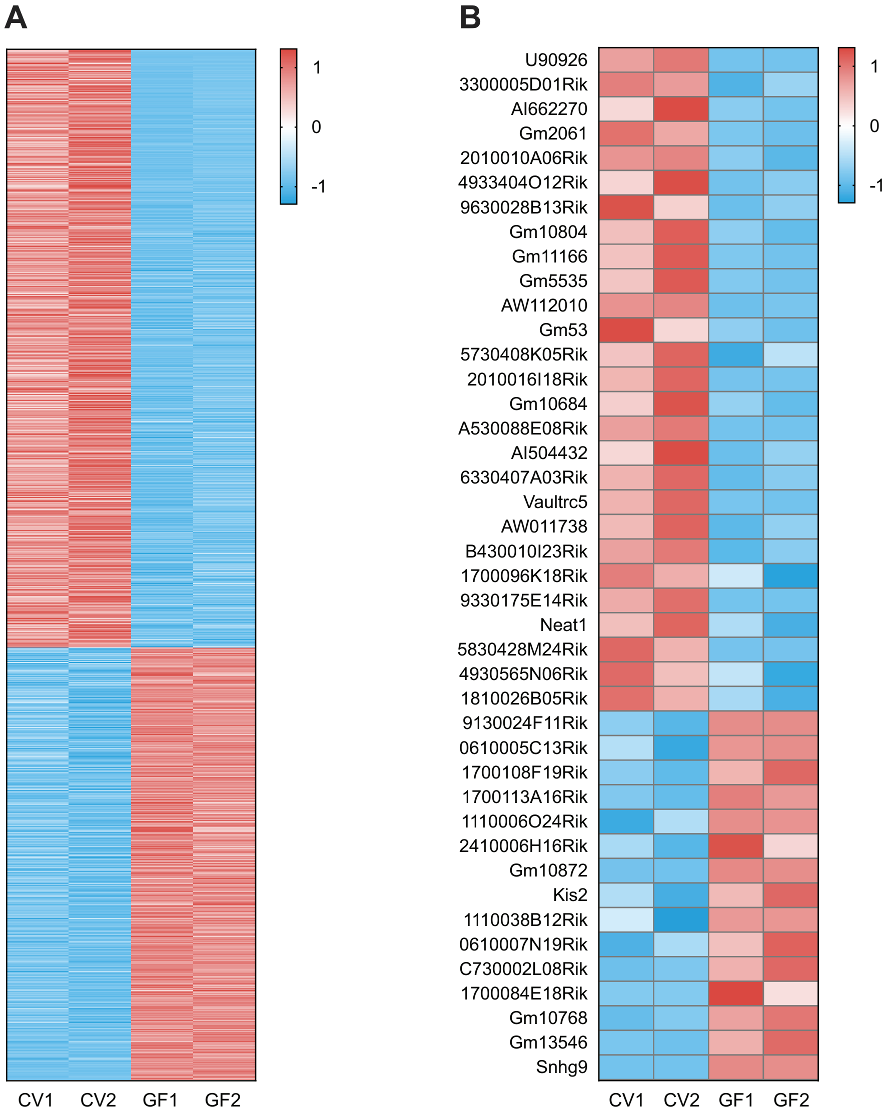

Fig. S1: Intestinal epithelial cells from germ-free mice have an altered transcriptome as compared to conventional mice.

Whole transcriptome sequencing of small intestinal epithelial cell transcripts in conventional (CV) and germ-free (GF) mice. **(A)** Heatmap visualizing genes that are differentially expressed between conventional and germ-free mice. **(B)** Heatmap visualizing lncRNA genes that are differentially expressed between conventional and germ-free mice.

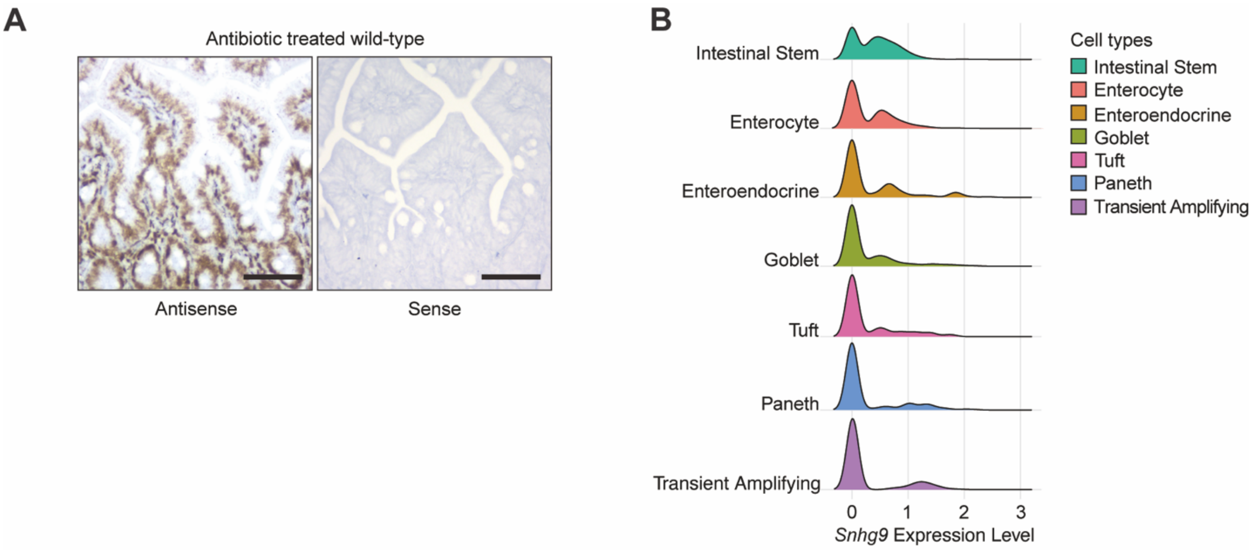

Fig. S2: *Snhg9* is expressed in intestinal epithelial cells.
(A) In situ hybridization of *Snhg9* in paraffin-embedded ileum sections from antibiotic-treated wild-type C57BL/6 mice. Scale bar = 50 µm. **(B)** *Snhg9* expression from a published single-cell RNA-seq analysis of mouse intestinal epithelial cells (*20*).

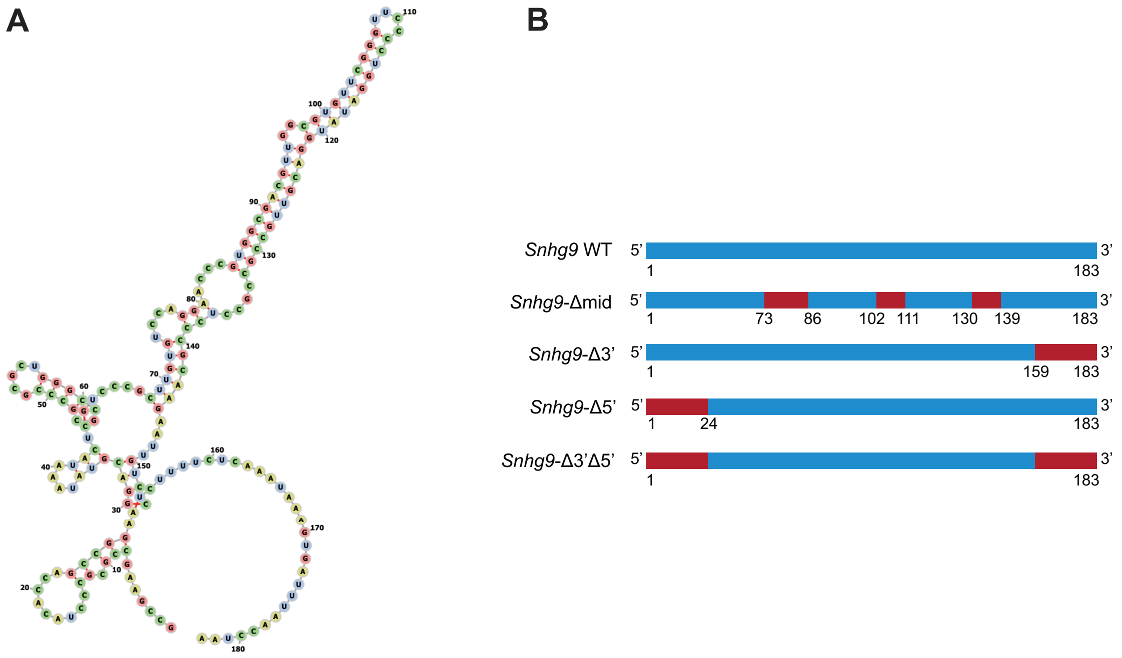

Fig. S3: Secondary structure of long non-coding RNA *Snhg9*.

(A) Secondary structure of *Snhg9* RNA predicted by RNAfold (*49*) with nucleotide positions indicated.

(B) Locations of nucleotide deletions analyzed in Fig. 2E.

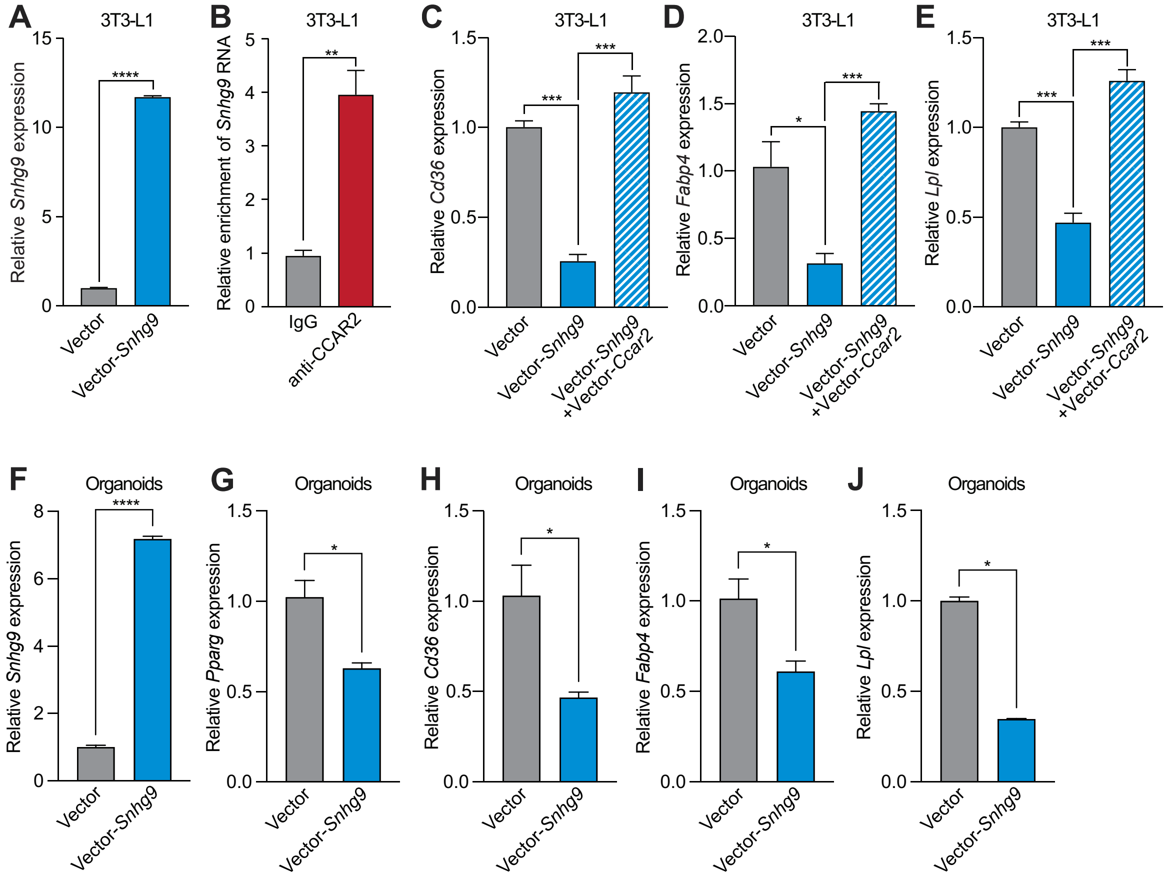

Fig. S4: Stable expression of *Snhg9* in 3T3-L1 cells and mouse intestinal organoids decreases expression of PPARγ-regulated genes.

(A) qPCR analysis of *Snhg9* expression in 3T3-L1 cells stably expressing *Snhg9*. N=4 experimental replicates per group. **(B)** RNA immunoprecipitation analysis of the binding between *Snhg9* RNA and CCAR2 in 3T3-L1 cells stably expressing *Snhg9*. *Snhg9* RNA precipitated by CCAR2 was detected by qPCR. N=3 experimental replicates per group. **(C to E)** qPCR analysis of *Cd36*, *Fabp4*, and *Lpl* in 3T3-L1 cells stably expressing *Snhg9* or co-expressing *Snhg9* and *Ccar2*, and in cells transduced with empty vector. N=4 experimental replicates per group. **(F to J)** qPCR analysis of *Snhg9*, *Pparg*, *Cd36*, *Fabp4*, and *Lpl* expression in mouse intestinal organoids stably expressing *Snhg9*. N=4 experimental replicates per group. Means ± SEM are plotted. *p<0.05; **p<0.01; ***p<0.001; ****p<0.0001; two-tailed Student's *t* test.

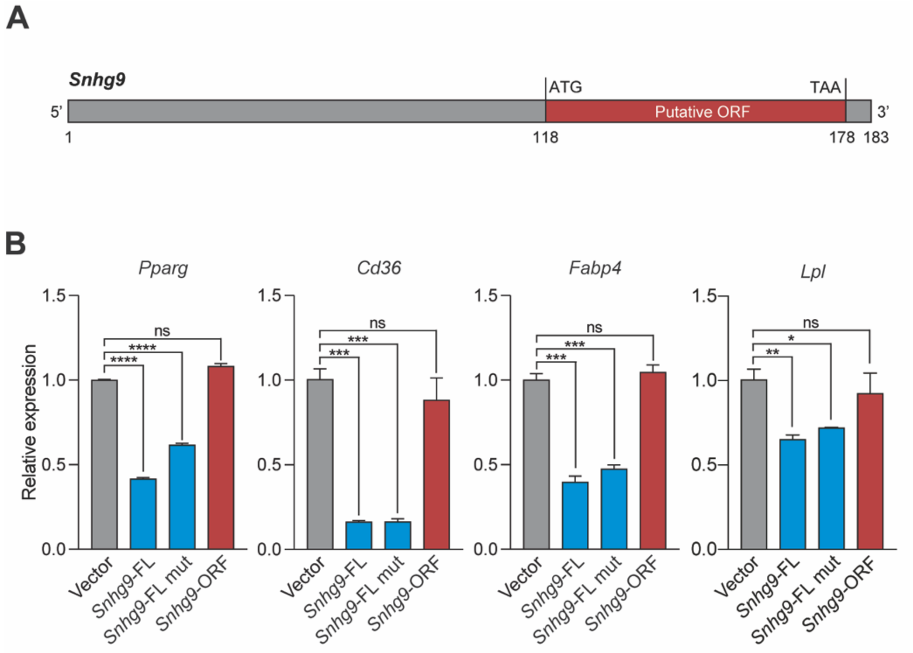

Fig. S5: Suppression of *Pparg* expression by *Snhg9* is not due to expression of the predicted open reading frame.

(A) Schematic diagram indicating the location of the predicted open reading frame (ORF). **(B)** Full-length *Snhg9* (*Snhg9*-FL), mutant full-length *Snhg9* (*Snhg9*-FL mut) with the start (ATG) and stop (TAA) codons mutated, and the *Snhg9* ORF were expressed in 3T3-L1 cells. Expression of *Pparg* and the downstream PPARγ target genes *Cd36*, *Fabp4*, and *Lpl* was assessed by qPCR. N=3 experimental replicates per group. Means ± SEM are plotted. *p<0.05; **p<0.01; ***p<0.001; ****p<0.0001; ns, not significant; two-tailed Student's *t* test.

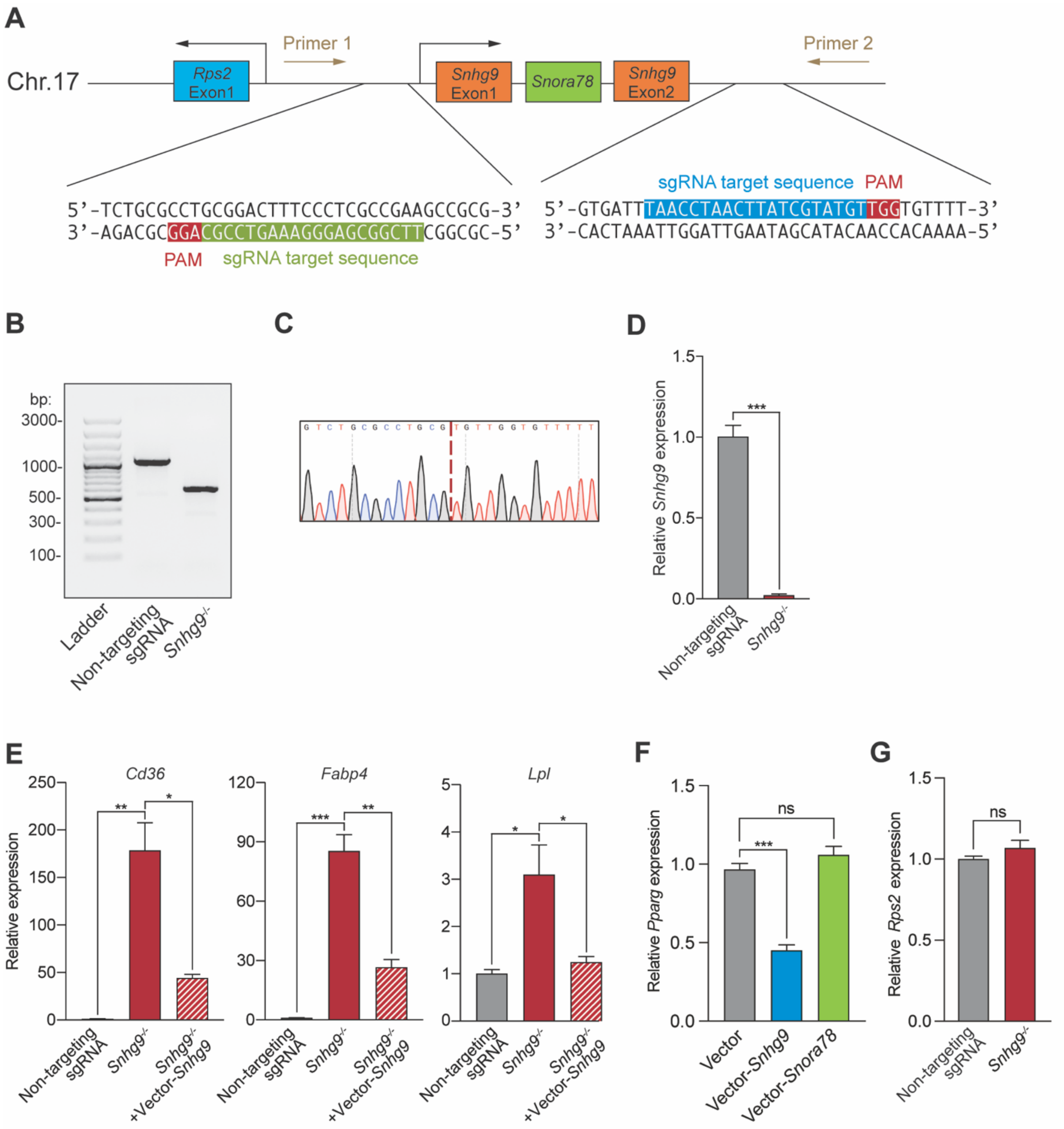

Fig. S6: Deletion of *Snhg9* in 3T3-L1 cells increases expression of PPARγ-regulated genes.
(A) Schematic diagram showing the CRISPR/Cas9-mediated *Snhg9* editing strategy. The single-stranded guide RNAs (sgRNA) specific for the *Snhg9* locus are shown. PAM, protospacer adjacent motif. **(B)** Genotyping of *Snhg9*$^{-/-}$ 3T3-L1 cells and cells edited with non-targeting sgRNA. **(C)** Sanger sequencing of the CRISPR/Cas9-edited region in the genomes of *Snhg9*$^{-/-}$ 3T3-L1 cells. Dashed red line indicates the cutting site. **(D)** qPCR analysis of *Snhg9* expression in *Snhg9*$^{-/-}$ 3T3-L1 cells and cells edited with non-targeting sgRNA. N=4 experimental replicates per group. **(E)** qPCR analysis of *Cd36*, *Fabp4*, and *Lpl* expression in *Snhg9*$^{-/-}$ 3T3-L1 cells that were untreated or rescued with *Snhg9* expression, and in cells edited with non-targeting sgRNA. N=4 experimental replicates per group. **(F)** qPCR analysis of *Pparg* expression in 3T3-L1 cells stably expressing *Snhg9* or *Snora78*. N=4 experimental replicates per group. **(G)** qPCR analysis of *Rps2* expression in *Snhg9*$^{-/-}$ 3T3-L1 cells and cells edited with non-targeting sgRNA. N=4

experimental replicates per group. Means ± SEM are plotted. *$p<0.05$; **$p<0.01$; ***$p<0.001$; ns, not significant; two-tailed Student's *t* test.

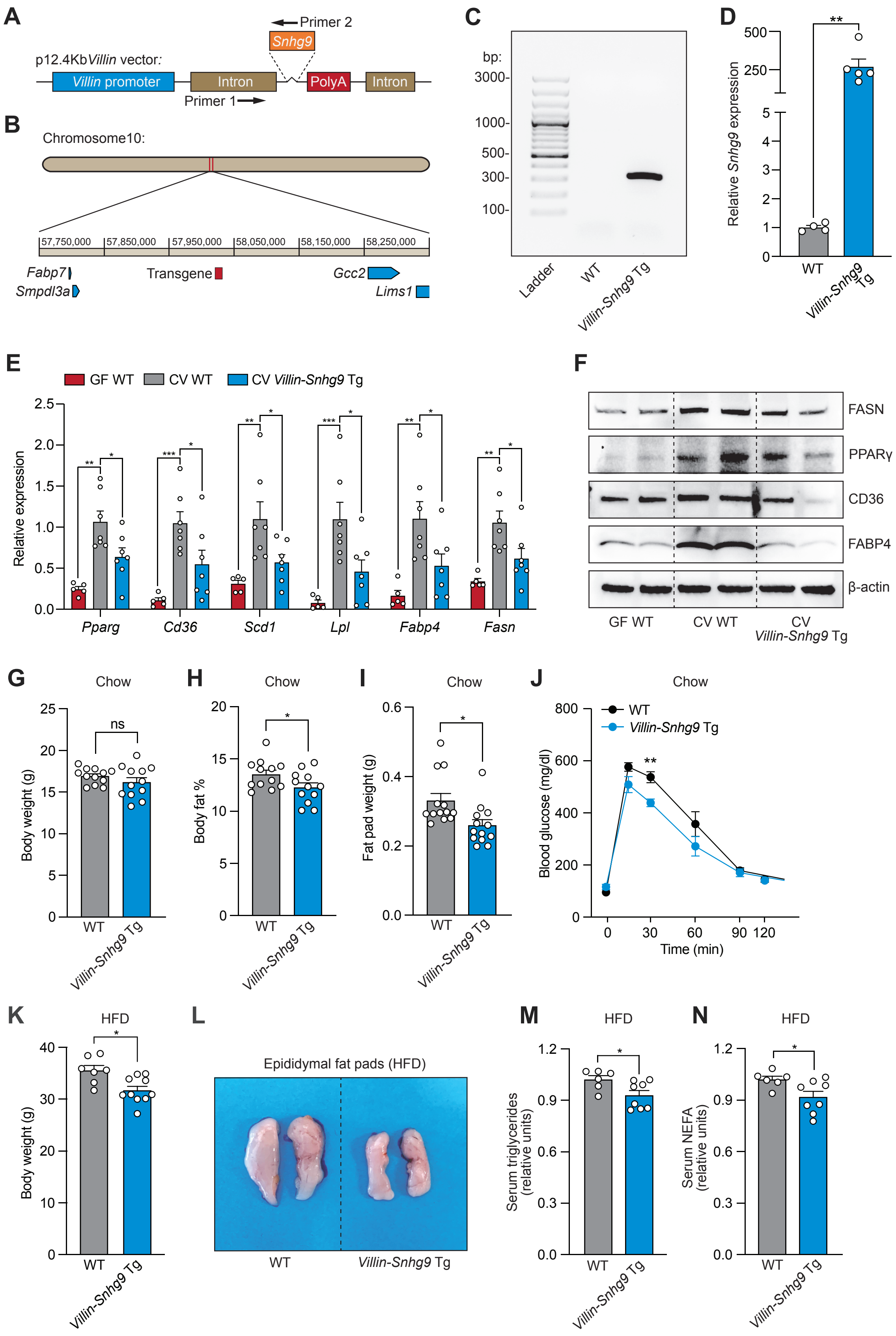
A
Primer 2
Snhg9
p12.4Kb Villin vector:
Villin promoter
Intron
PolyA
Intron
Primer 1
B
Chromosome10:
57,750,000
57,850,000
57,950,000
58,050,000
58,150,000
58,250,000
Fabp7
Smpdl3a
Transgene
Gcc2
Lims1
C
bp:
3000-
1000-
500-
300-
100-
Ladder
WT
Villin-Snhg9 Tg
D
**
500
250
5
4
3
2
1
0
Relative Snhg9 expression
WT
Villin-Snhg9 Tg
E
GF WT
CV WT
CV Villin-Snhg9 Tg
Relative expression
2.5
2.0
1.5
1.0
0.5
0.0
Pparg
Cd36
Scd1
Lpl
Fabp4
Fasn
F
FASN
PPARγ
CD36
FABP4
β-actin
GF WT
CV WT
CV Villin-Snhg9 Tg
G
Chow
ns
Body weight (g)
25
20
15
10
5
0
WT
Villin-Snhg9 Tg
H
Chow
*
Body fat %
20
15
10
5
0
WT
Villin-Snhg9 Tg
I
Chow
*
Fat pad weight (g)
0.6
0.4
0.2
0.0
WT
Villin-Snhg9 Tg
J
Chow
WT
Villin-Snhg9 Tg
**
Blood glucose (mg/dl)
800
600
400
200
0
0
30
60
90
120
Time (min)
K
HFD
*
Body weight (g)
40
30
20
10
0
WT
Villin-Snhg9 Tg
L
Epididymal fat pads (HFD)
WT
Villin-Snhg9 Tg
M
HFD
*
Serum triglycerides (relative units)
1.2
0.9
0.6
0.3
0.0
WT
Villin-Snhg9 Tg
N
HFD
*
Serum NEFA (relative units)
1.2
0.9
0.6
0.3
0.0
WT
Villin-Snhg9 Tg

Fig. S7: *Villin-Snhg9* transgenic mice show lowered expression of lipid metabolic genes and reduced body fat.

(A) Schematic diagram of the *Villin-Snhg9* transgene vector. **(B)** Schematic diagram of the *Villin-Snhg9* transgene insertion site. Whole genome sequencing revealed that the transgene was integrated into an intergenic region between the genes *Smpdl3a* and *Gcc2*. **(C)** Genotyping of wild-type and *Villin-Snhg9* transgenic mice. **(D)** qPCR analysis of *Snhg9* expression in the intestines of wild-type and *Villin-Snhg9* transgenic littermates. Each data point represents one mouse. **(E)** qPCR analysis of *Pparg*, *Cd36*, *Scd1*, *Lpl*, *Fabp4* and *Fasn* expression in the small intestines of germ-free, wild-type and *Villin-Snhg9* transgenic littermates. Each data point represents one mouse. **(F)** Immunoblot detection of PPARγ, CD36, FABP4 and FASN in small intestines of germ-free (GF) wild-type (WT), conventional wild-type and conventional *Villin-Snhg9* transgenic littermates. β-actin was detected as the loading control. **(G)** Body weights of 10 week-old conventional wild-type and *Villin-Snhg9* transgenic littermates fed a chow diet. Each data point represents one mouse. **(H)** Body fat percentages of 10 week-old conventional wild-type and *Villin-Snhg9* transgenic littermates fed a chow diet. Each data point represents one mouse. **(I)** Epididymal fat pad weight of 10 week-old conventional wild-type and *Villin-Snhg9* transgenic littermates fed a chow diet. Each data point represents one mouse. **(J)** Glucose tolerance test on 10 week-old conventional wild-type and *Villin-Snhg9* transgenic littermates fed a chow diet. N=5 mice per group. **(K)** Body weights of wild-type (WT) and *Villin-Snhg9* transgenic mice fed a high fat diet for 10 weeks. Each data point represents one mouse. **(L)** Representative image of epididymal fad pads of wild-type and *Villin-Snhg9* transgenic mice. **(M and N)** Relative serum triglycerides (L) and non-esterified fatty acids (NEFA) (M) concentrations in wild-type and *Villin-Snhg9* transgenic mice fed a high fat diet for 10 weeks. Each data point represents one mouse. Means ± SEM are plotted. *p<0.05; **p<0.01; ***p<0.001; ns, not significant; two-tailed Student's *t* test.

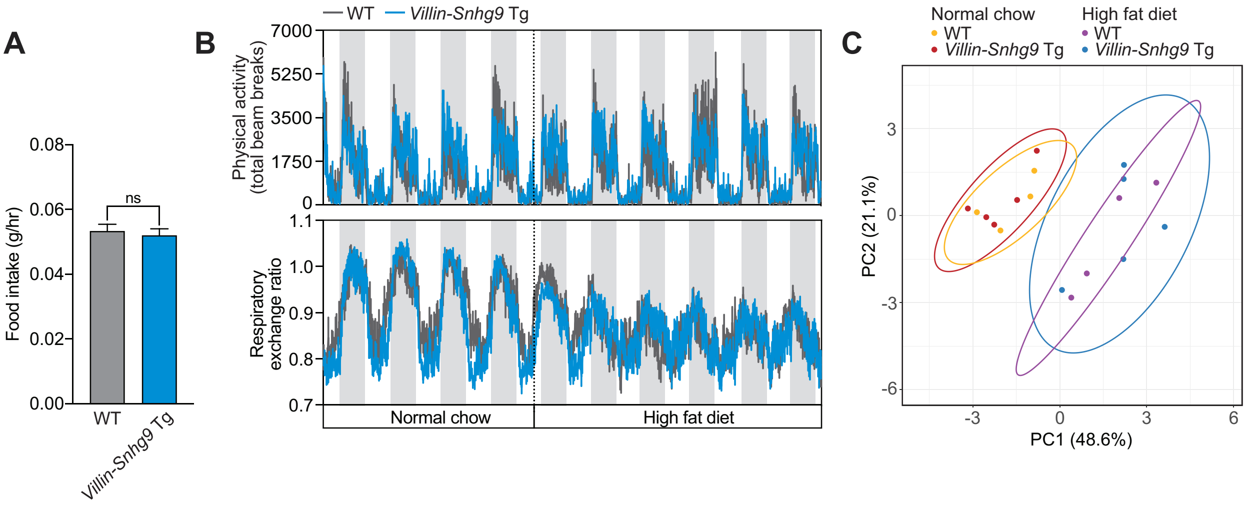

Fig. S8: Food intake, physical activity and intestinal microbiota composition of *Villin-Snhg9* transgenic mice.

(A) Food intake rate for wild-type (WT) and *Villin-Snhg9* transgenic mice. N=6 mice per group. **(B)** Recording of total physical activity (upper panel) and respiratory exchange ratio (lower panel) of wild-type and *Villin-Snhg9* transgenic mice over 10 days. The diet was switched from normal chow to a high fat diet at day 5. N=6 mice per group. **(C)** Principal coordinate (PC) analysis of 16*S* rRNA sequencing of fecal samples from *Villin-Snhg9* transgenic mice and co-housed wild-type (WT) littermates. Fecal samples were collected and sequenced before and after switching from normal chow to a high fat diet. Each data point represents one mouse. Means ± SEM are plotted. ns, not significant; two-tailed Student's *t* test.

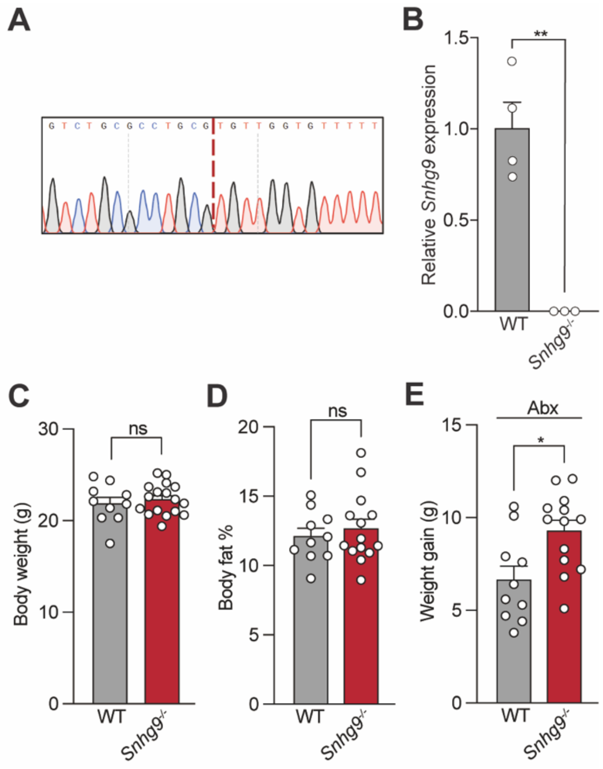

Fig. S9: Antibiotic-treated *Snhg9*$^{-/-}$ mice show increased weight gain relative to wild-type mice when fed a high fat diet.

(A) Sanger sequencing of the CRISPR/Cas9-edited region of *Snhg9*$^{-/-}$ mice. Dashed red line indicates the edited site. The editing strategy is the same as shown in fig. S6A for 3T3-L1 cells. **(B)** qPCR analysis of *Snhg9* expression in *Snhg9*$^{-/-}$ and wild-type littermates. Each data point represents one mouse. **(C and D)** Body weight and body fat percentage of *Snhg9*$^{-/-}$ and wild-type littermates fed a chow diet. Each data point represents one mouse. **(E)** Weight gain of *Snhg9*$^{-/-}$ and wild-type littermates treated with antibiotics (Abx) and fed a high fat diet for 10 weeks. Each data point represents one mouse. Means ± SEM are plotted. *p<0.05; **p<0.01; ns, not significant; two-tailed Student's *t* test.

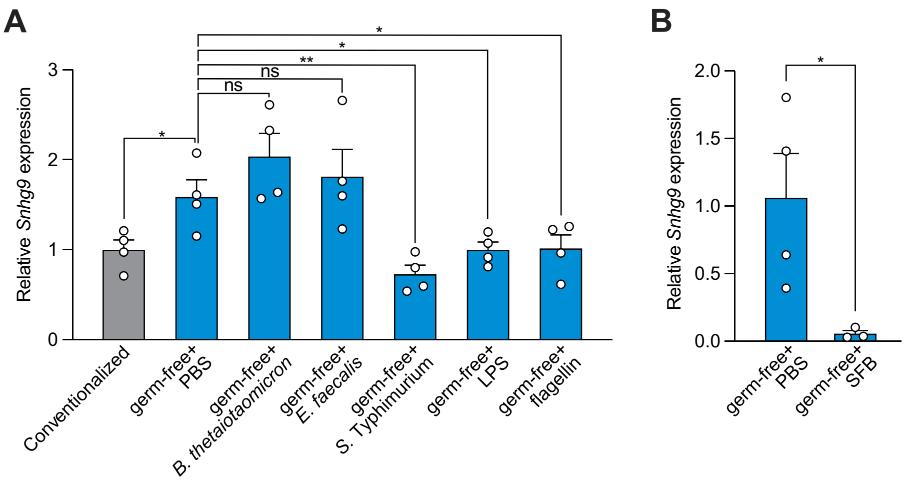

Fig. S10: *Salmonella* Typhimurium and segmented filamentous bacteria repress *Snhg9* expression. **(A)** qPCR analysis of *Snhg9* expression in germ-free mice transplanted with feces from conventional mice (conventionalized), treated with PBS, monocolonized with *B. thetaiotaomicron*, *E. faecalis* or *S.* Typhimurium, or treated with LPS or flagellin. Each data point represents one mouse. **(B)** qPCR analysis of *Snhg9* expression in germ-free mice treated with PBS or monocolonized with SFB. Each data point represents one mouse. Means ± SEM are plotted. *p<0.05; **p<0.01; ns, not significant; two-tailed Student's *t* test.

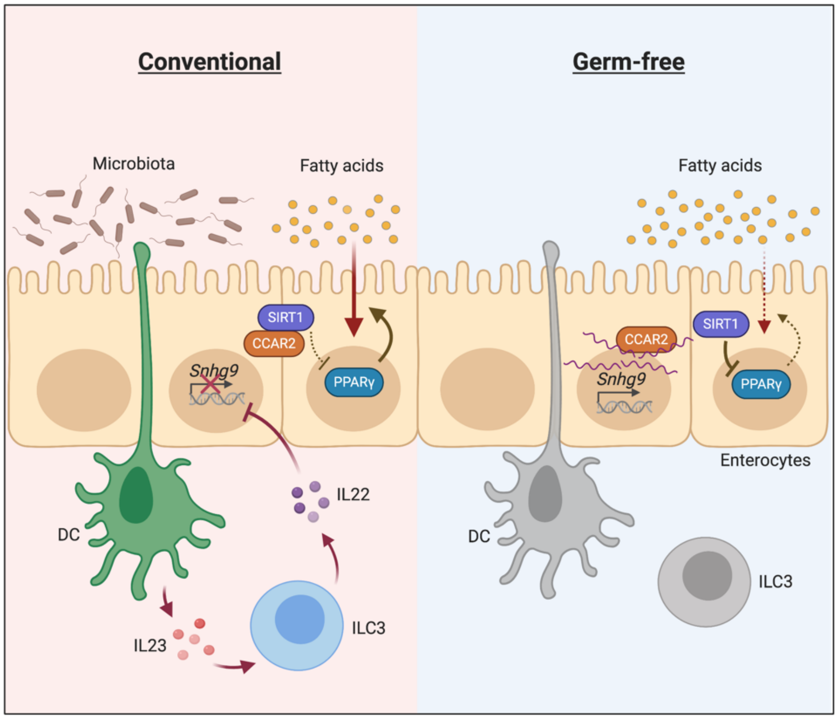

Fig. S11: Model of lncRNA *Snhg9* regulation of lipid metabolism in the intestine.
In conventional mice, expression of *Snhg9* is repressed by the microbiota through a myeloid cell-ILC3 signaling relay. In the absence of *Snhg9* RNA, CCAR2 and SIRT1 remain bound and PPARγ promotes the expression of lipid metabolic genes. In germ-free mice, the absence of a microbiota leads to elevated expression of *Snhg9*. *Snhg9* RNA binds to CCAR2 and promotes its dissociation from SIRT1. Free SIRT1 inhibits PPARγ activity and leads to reduced lipid metabolism in the intestine. Created with BioRender.com

**Primer Sequences for qPCR**		
Snhg9	Forward	5'- CCAGCCGGAAGGACGTATAA -3'
	Reverse	5'- CACGGGTTCCTGGACACAAG -3'
Pparg	Forward	5'- GTGATGGAAGACCACTCGCATT -3'
	Reverse	5'- CCATGAGGGAGTTAGAAGGTTC -3'
Cd36	Forward	5'- TCATATTGTGCTTGCAAATCCAA -3'
	Reverse	5'- TGTAGATCGGCTTTACCAAAGATG -3'
Lpl	Forward	5'- CCAATGGAGGCACTTTCCAG -3'
	Reverse	5'- CCACGTCTCCGAGTCCTCTC -3'
Fabp4	Forward	5'- TCACCGCAGACGACAGGAA -3'
	Reverse	5'- CCACCAGCTTGTCACCATCTC -3'
Scd1	Forward	5'- CTTCTTCTCTCACGTGGGTTG -3'
	Reverse	5'- CGGGCTTGTAGTACCTCCTC -3'
Fasn	Forward	5'- TCCTGGAACGAGAACACGATCT -3'
	Reverse	5'- GAGACGTGTCACTCCTGGACTTG -3'
Rps2	Forward	5'- CGCGCTTCTTGGAGCACTA -3'
	Reverse	5'- GTCATCCGCCATTTGGTGTT -3'
**Sequences of sgRNAs used to generate *Snhg9*$^{-/-}$ 3T3-L1 cells**		
Snhg9 locus	Front	5'- TTCGGCGAGGGAAAGTCCGC -3'
	Rear	5'- TAACCTAACTTATCGTATGT -3'
Non-targeting	-	5'- GCGAGGTATTCGGCTCCGCG -3'
**Genotyping primers**		
Villin-Snhg9 Tg mice	Forward	5'- GCTGGGCTGTGTAACAGGCAC -3'
	Reverse	5'- CAACGTCGCCACGGGTTCCTG -3'
Snhg9$^{-/-}$ mice and 3T3-L1 cells	Forward	5'- GCCGCGGAAGGGACAATTTA -3'
	Reverse	5'- TTTAGCGTAGGTGGTCATCTTTT -3'

Table S1: Sequences of the oligonucleotides used in the manuscript.
